# General Perturbation Resilient Dynamic String-Averaging for Inconsistent Problems with Superiorization

**DOI:** 10.1007/s10957-025-02763-9

**Published:** 2025-07-08

**Authors:** Kay Barshad, Yair Censor

**Affiliations:** Department of Mathematics, University of Haifa, Mt. Carmel, Haifa, 3498838 Israel

**Keywords:** Approximately shrinking operator, Bounded perturbation resilience, Bounded regularity, Coherence, Coherent sequence of operators, Common fixed point problem, Convex feasibility problem, Dynamic string-averaging, Fejér monotonicity, Metric projection, Nonexpansive operator, Strong coherence, Strongly coherent sequence of operators, Superiorization, Weak convergence, Weak regularity, 46N10, 46N40, 47H09, 47H10, 47J25, 47N10, 65F10, 65J99

## Abstract

In this paper we introduce a General Dynamic String-Averaging (GDSA) iterative scheme and investigate its convergence properties in the inconsistent case, that is, when the input operators don’t have a common fixed point. The Dynamic String-Averaging Projection (DSAP) algorithm itself was introduced in an 2013 paper, where its strong convergence and bounded perturbation resilience were studied in the consistent case (that is, when the sets under consideration had a nonempty intersection). Results involving combination of the DSAP method with superiorization, were presented in 2015. The proof of the weak convergence of our GDSA method is based on the notion of “strong coherence” of sequences of operators that was introduced in 2019. This is an improvement of the property of “coherence” of sequences of operators introduced in 2001 by Bauschke and Combettes. Strong coherence provides a more convenient sufficient convergence condition for methods that employ infinite sequences of operators and it turns out to be a useful general tool when applied to proving the convergence of many iterative methods. In this paper we combine the ideas of both dynamic string-averaging and strong coherence, in order to analyze our GDSA method for a general class of operators and its bounded perturbation resilience in the inconsistent case with weak and strong convergence. We then discuss an application of the GDSA method to the Superiorization Methodology, developing results on the behavior of its superiorized version.

## Introduction

A strongly convergent Dynamic String-Averaging Projection (DSAP) algorithm, based on convex combinations and compositions of operators, was introduced, along with its bounded perturbation resilience, by Censor and Zaslavski in [22] for a family of nonempty, closed and convex sets $\left\{ C_{i}\right\} _{i=1}^{m}$, where the considered operators were given by a sequence of metric projections $\left\{ P_{C_{i}}\right\} _{i=1}^{m}$ in the consistent case, that is, when $\cap _{i=1}^{m}C_{i}\not =\emptyset$. A superiorized version of this algorithm appeared in [23], where it was proved that any sequence generated by it either converges to a constrained minimum point of the objective function employed in the superiorization process, or that it is strictly Fejér monotone with respect to a subset of the solution set of the constrained minimization problem. String-averaging projection (SAP) methods form a general algorithmic framework introduced in [19]. Subsequently, they were developed in a variety of situations such as for convex feasibility with infinitely many sets [27], for incremental stochastic subgradient algorithms [24] and for proton computed tomography image reconstruction [6], to name but a few. See also [2], where perturbation resilience of such methods was further studied and [1], where linear convergence rates for a certain class of extrapolated fixed point algorithms which are based on dynamic string-averaging methods were established.

In the present paper we consider more general ideas, focusing on more general algorithmic structures which are applied to the inconsistent case (that is, when the input operators don’t have a common fixed point), and when the objective function, involved in the superiorization, is only required to be convex and continuous. For a positive integer *m* and a given family of nonexpansive operators $\left\{ U_{i}\right\} _{i=1}^{m}$, $U_{i}:\mathcal {H}\rightarrow \mathcal {H}$ for each $i=1,2,\dots ,m$, without a common fixed point, we construct a General Dynamic String-Averaging (GDSA) algorithm (Algorithm 4.5 below) based on convex combinations, compositions and relaxations of the operators of the aforementioned family. In Theorem 4.8 below we investigate weak and strong convergence properties of our GDSA algorithm and its bounded perturbation resilience. To this end we introduce a “$\limsup$-admissible sequence of operators” (Definition 4.2 below) and show that if, after a certain GDSA procedure, the sequence of output operators is $\limsup$-admissible, then the algorithm converges weakly (and under additional assumptions – strongly) to a point in a certain (assumed nonempty) set, even though the given input operators don’t have a common fixed point.

We further apply our GDSA algorithm to the Superiorization Methodology (SM). The SM is not aiming to solve the constrained minimization problem under consideration, but to find a feasible point for the original feasibility-seeking problem which is “superior”, i.e., has smaller or equal objective function value than that of a point returned by the feasibility-seeking only algorithm that is employed by the SM. More details about the SM appear below in Subsection 5.1.

The weak convergence of our GDSA algorithm and its superiorized version is based, inter alia, on the theory of coherence, which was introduced by Bauschke and Combettes in [9] and further developed by Barshad, Reich and Zalas in [5], see also [3] and [4].

All the results below, concerning our algorithmic schemes, remain valid in the consistent case as well. However, since it is generally desirable to assume certain properties of the input operators, which is possible in this case as it was shown in [29], such results are of limited interest in the consistent case due to the assumptions placed on the output operators in our GDSA procedure.

It is worth noting the significance of the inconsistent case at this point. The convex feasibility problem (CFP) is to find a feasible point in the intersection of a family of convex and closed sets. If the intersection is empty, then the CFP is inconsistent and a feasible point does not exist. However, algorithmic research on inconsistent CFPs does exist and is mainly focused on two directions. One is oriented toward defining other solution concepts that will apply, such as proximity function minimization, wherein a proximity function measures, in some way, the total violation of all constraints (see, for instance, Example 4.10 below). The second direction investigates the behavior of algorithms that are designed to solve a consistent CFP when applied to inconsistent problems. This direction is fueled by situations wherein one lacks a priori information about the consistency or inconsistency of the CFP or does not wish to invest computational resources to get hold of such knowledge prior to running his algorithm. A telegraphical review of some recent works on inconsistent CFPs appears in [21].

The rest of the paper is organized as follows. In Section 2 we provide some background which is needed to establish our results. In Section 3 we introduce the general bounded regularity with its properties and discuss the well-known notion of approximate shrinking of operators. In Section 4 we present the properties of our GDSA algorithm. Notations and initial tools appear in Subsection 4.1 and lemmata leading to the main result are in Subsection 4.2. In Section 5, we present applications of this algorithm to the Superiorization Methodology, followed by a brief summary with conclusions in Section 6.

## Preliminaries

Throughout this paper $\mathbb {N}$ denotes the set of natural numbers (starting from 0), and for any two integers *m* and *n*, with $m\le n$, we denote by $\left\{ m,m+1,\dots ,n\right\}$ the set of all integers between *m* and *n*. The real line is denoted by $\mathbb {R}$. For a set *A*, we denote by $\left| A\right|$ the cardinality of *A*. For a real Hilbert space $\mathcal {H}$, we use the following notations:

- $\langle \cdot ,\cdot \rangle$ denotes the inner product on $\mathcal {H}.$
- $\Vert \cdot \Vert$ denotes the norm on $\mathcal {H}$ induced by $\langle \cdot ,\cdot \rangle .$
- *Id* denotes the identity operator on $\mathcal {H}$.
- $\textrm{Fix}T$ denotes the, possibly empty, set $\textrm{Fix}T:=\{x\in \mathcal {H}\mid T(x)=x\}$ of fixed points of an operator $T:\mathcal {H}\rightarrow \mathcal {H}$.
- For a nonempty and convex subset *C* of $\mathcal {H}$, we denote by $P_{C}$ the (unique) metric projection onto *C*, the existence of which is guaranteed if *C* is, in addition, closed.
- The expressions $x^{k}\rightharpoonup x$ and $x^{k}\rightarrow x$ denote, the weak and strong convergence, respectively, to *x* of a sequence $\left\{ x^{k}\right\} _{k=0}^{\infty }$ in $\left( \mathcal {H},\Vert \cdot \Vert \right)$ when $k\rightarrow \infty$, while $\mathfrak {W}\left( \left\{ x^{k} \right\} _{k=0}^{\infty }\right)$ denotes the set of weak cluster points of $\left\{ x^{k}\right\} _{k=0}^{\infty }$.
- For a convex function $\phi :\mathcal {H}\rightarrow \mathbb {R}$ and a point $x\in \mathcal {H}$, we denote by $\partial \phi \left( x\right)$ the subdifferential of $\phi$ at *x*, that is,
  2.1 $$\begin{aligned} \partial \phi \left( x\right) :=\left\{ g\in \mathcal {H}\,|\,\left\langle g,y-x\right\rangle \le f\left( y\right) -f\left( x\right) \,\,\textrm{for}\,\,\textrm{all}\,\,y\in \mathcal {H}\right\} . \end{aligned}$$
- For a function $f:\mathcal {H}\rightarrow \mathbb {R}$ and a subset *A* of $\mathcal {H}$, we denote by $\underset{x\in A}{\textrm{Argmin}}f\left( x\right)$ the set of minimizers of *f* on the set *A*.
- $B\left( x,r\right)$ denotes the open ball centered at $x\in \mathcal {H}$ of radius $r>0$.
- For a nonempty subset *C* of $\mathcal {H}$ and $x\in \mathcal {H}$, we denote by $d\left( x,C\right)$ the distance from *x* to *C*, that is, $d\left( x,C\right) :=\inf _{y\in C}\left\| x-y\right\|$.

We recall the following types of algorithmic operators. For more information on such operators, see, for example, [13].

### Definition 2.1

Let $T:\mathcal {H}\rightarrow \mathcal {H}$ be an operator and let $\lambda \in \left[ 0,2\right]$. The operator $T_{\lambda }:\mathcal {H}\rightarrow \mathcal {H}$ defined by $T_{\lambda }:=\left( 1-\lambda \right) {Id}+\lambda T$ is called a $\lambda$-*relaxation* of the operator *T*. The operator $T_{2}$ is called a *reflection* of the operator *T*.

### Definition 2.2

An operator $T:\mathcal {H}\rightarrow \mathcal {H}$ is said to be *nonexpansive* (NE) if

$$ \left( \forall x,y\in \mathcal {H}\right) \,\,\left\| T\left( x\right) -T\left( y\right) \right\| \le \left\| x-y\right\| . $$

For $\lambda \in \left[ 0,2\right]$, an operator $T:\mathcal {H}\rightarrow \mathcal {H}$ is said to be $\lambda$-*relaxed nonexpansive* if *T* is a $\lambda$-relaxation of a nonexpansive operator *U*, that is, $T=U_{\lambda }$.

### Remark 2.3

Clearly, convex combinations and compositions of nonexpansive operators are also nonexpansive. Moreover, for each $\lambda \in \left[ 0,1\right]$, the $\lambda$-relaxation of a nonexpansive operator is also nonexpansive.

A central role in our analysis is played by cutter operators that we define next. For every ordered pair $\left( x,y\right) \in \mathcal {H}^{ 2 }$, we define the closed and convex set $H\left( x,y\right)$ by

$$ H\left( x,y\right) :=\left\{ u\in \mathcal {H}\,\vert \,\langle u-y,x-y\rangle \le 0\right\} . $$

The following class of cutters was introduced by Bauschke and Combettes in [9], with a different terminology and named there the “class ${\mathfrak {T}}$”. The name “cutter” was proposed in [14]; other names are used in the literature for these operators, such as, e.g., “firmly quasi-nonexpansive” (see, for example, Definition 4.1 and Proposition 4.2 in [10]), where various properties and examples of cutters can be found.

### Definition 2.4

An operator $T:\mathcal {H}\rightarrow \mathcal {H}$ is called a *cutter* if it satisfies the condition

$$ \text {Fix}T\subseteq H\left( x,T\left( x\right) \right) , \forall x,y\in X, $$

or, equivalently, $\left\langle z-T(x),x-T(x)\right\rangle \le 0$ for each $z\in \textrm{Fix}T$ and $x\in X$. For $\lambda \in \left[ 0,2\right]$, an operator $T:\mathcal {H}\rightarrow \mathcal {H}$ is a $\lambda$-*relaxed *cutter if *T* is a $\lambda$-relaxation of a cutter *U*, that is, $T=U_{\lambda }=\left( 1-\lambda \right) \textit{Id}+\lambda U$.

### Proposition 2.5

Let *T* be a cutter. Then $\textrm{Fix}T=\cap _{x\in \mathcal {H}}H\left( x,T\left( x\right) \right)$ and hence $\textrm{Fix}T$ is a closed and convex subset of $\mathcal {H}$, as an intersection of half-spaces.

### Proof

See Proposition 2.6 in [9]. $\square$

### Remark 2.6

It is worthwhile to caution the reader about some ambiguity in the literature regarding the term cutter. Definition 9.2 of [14] is the first original definition of cutters and they are defined there without any condition on the nonemptiness of the fixed points set of the operators. Cutters are, thus, just another name for the members of the original “class ${\mathfrak {T}}$” of Bauschke and Combettes in [9] which are also defined there without any condition on the nonemptiness of the fixed points set of the operators. However, in Definition 2.1.30 in [13] the condition that the fixed points set of the cutter operators should be nonempty was included in the definition. This created some ambiguity due to the fact that some later publications either include or do not include the condition that the fixed points set of the cutter operators should be nonempty.

We adhere to the original definition (Definition 9.2 of [14]) and when we need the fixed points set of the cutter to be nonempty we explicitly say so.

### Remark 2.7

Clearly, $\textrm{Fix}T=\textrm{Fix}T_{\lambda }$ for every operator $T:\mathcal {H}\rightarrow \mathcal {H}$ and every $\lambda \in \left( 0,2\right]$. Moreover, if *T* is a cutter, then for every $\lambda \in \left[ 0,1\right] ,$ $T_{\lambda }$ is also a cutter since $H\left( x,Tx\right) \subset H\left( x,T_{\lambda }\left( x\right) \right)$ for each $x\in \mathcal {H}$ and each $\lambda \in \left[ 0,1\right]$.

### Definition 2.8

We say that an operator $T:{\mathcal {H}}\rightarrow \mathcal {H}$ is:

(i) *Firmly nonexpansive *(FNE) if
  $$ \left( \forall x,y\in \mathcal {H}\right) \left\langle T\left( x\right) -T\left( y\right) ,x-y\right\rangle \ge \left\| T\left( x\right) -T\left( y\right) \right\| ^{2}. $$
(ii) $\rho$-*firmly nonexpansive* ($\rho$-FNE), where $\rho \ge 0$ is a real number, if
  $$ \left( \forall x,y\in \mathcal {H}\right) \left\| T\left( x\right) -T\left( y\right) \right\| ^{2}\le \left\| x-y\right\| ^{2}-\rho \left\| \left( x-T\left( x\right) \right) -\left( y-T\left( y\right) \right) \right\| ^{2}. $$

### Definition 2.9

For $\lambda \in \left[ 0,2\right]$, an operator $T:\mathcal {H}\rightarrow \mathcal {H}$ is called $\lambda$-*relaxed firmly nonexpansive* if *T* is a $\lambda$-relaxation of a firmly nonexpansive operator *U*, that is, $T=U_{\lambda }=\left( 1-\lambda \right) \textit{Id}+\lambda U$.

### Theorem 2.10

If $T:{\mathcal {H}}\rightarrow \mathcal {H}$ is firmly nonexpansive, then *T* is a nonexpansive cutter.

### Proof

See Theorem 2.2.4 and Theorem 2.2.5 in [13]. $\square$

### Theorem 2.11

Let $T:\mathcal {H}\rightarrow \mathcal {H}$ be an operator. The following conditions are equivalent:

(i) *T* is firmly nonexpansive.
(ii) $T_{\lambda }$ is nonexpansive for each $\lambda \in \left[ 0,2\right]$.
(iii) There exists a nonexpansive operator $N:\mathcal {H\rightarrow \mathcal {H}}$ such that $T=2^{-1}\left( Id+N\right)$.

### Proof

See Theorem 2.2.10 in [13]. $\square$

### Theorem 2.12

For any $\alpha \in \left( 0,2\right]$, an operator $T:\mathcal {H}\rightarrow \mathcal {H}$ is firmly nonexpansive if and only if its relaxation $T_{\alpha }$ is $\left( 2-\alpha \right) \alpha ^{-1}$-firmly nonexpansive, that is, if and only if

$$ \left( \forall x,y\in \mathcal {H}\right) \left\| T_{\alpha }\left( x\right) -T_{\alpha }\left( y\right) \right\| ^{2}$$

$$\le \left\| x-y\right\| ^{2}-\left( 2-\alpha \right) \alpha ^{-1}\left\| \left( x-T_{\alpha }\left( x\right) \right) -\left( y-T_{\alpha }\left( y\right) \right) \right\| ^{2}. $$

### Proof

See Corollary 2.2.15 in [13]. $\square$

### Corollary 2.13

Let $U:\mathcal {H}\rightarrow \mathcal {H}$ be an operator and let $T{:=}Id+2^{-1}\left( 1+\rho \right) \left( U-Id\right)$ for some $\rho \ge 0$, that is, $T=U_{2^{-1}\left( 1+\rho \right) }$. Then *U* is $\rho$-firmly nonexpansive if and only if *T* is firmly nonexpansive. In particular, *U* is nonexpansive if and only if $T:=\frac{1}{2}\left( U+Id\right)$ is firmly nonexpansive.

### Proof

Immediate from Theorem 2.12. $\square$

### Definition 2.14

We say that an operator $T:\mathcal {H}\rightarrow \mathcal {H}$ is:

(i) *Quasi-nonexpansive* if
  $$ \left\| T\left( x\right) -z\right\| \le \left\| x-z\right\| $$
  for each $x\in \mathcal {H}$ and $z\in \textrm{Fix}T$.
(ii) $\rho$*-strongly quasi-nonexpansive* for some $0\le \rho \in \mathbb {R}$ if
  2.2 $$\begin{aligned} \left\| T\left( x\right) -z\right\| ^{2}\le \left\| x-z\right\| ^{2}-\rho \left\| T\left( x\right) -x\right\| ^{2} \end{aligned}$$
  for all $x\in \mathcal {H}$ and for all $z\in \textrm{Fix}T$. If *T* satisfies (2.2) for some $\rho >0$, then it is called strongly quasi-nonexpansive.

### Remark 2.15

Clearly, for any $\rho \ge 0$, a $\rho$-strongly quasi-nonexpansive operator is, in particular, quasi-nonexpansive and a $\rho$-firmly nonexpansive operator operator is, in particular, $\rho$-strongly quasi-nonexpansive.

### Remark 2.16

Similarly to Remark 2.6 about cutters, we mention that in the literature (as, for example, Definitions 2.1.19 and 2.1.38 in [13]) quasi-nonexpansive and strongly quasi-nonexpansive operators are required to have a nonempty fixed point set by definition. Here we define these operators without this requirement and assume the nonemptiness of their fixed points set only if we need it.

### Corollary 2.17

For a positive integer *m*, let $\left\{ U_{i}\right\} _{i=1}^{m}$ be a finite family of $\rho _{i}$-firmly nonexpansive operators, where $U_{i}:\mathcal {H\rightarrow \mathcal {H}}$ and $\rho _{i}\in \left[ 0,\infty \right)$ for each $i=1,2,\dots ,m$. Set $\rho :=\min _{i\in \left\{ 1,2,\dots ,m\right\} }\rho _{i}$. Then:

(i) For each finite family of numbers $\left\{ \omega _{i}\right\} _{i=1}^{m}\subset \left[ 0,1\right]$ such that $\sum _{i=1}^{m}\omega _{i}=1$, the convex combination $U:=\sum _{i=1}^{m}\omega _{i}U_{i}$ is $\rho$-firmly nonexpansive.
(ii) The composition $V:=U_{m}\cdots U_{2}U_{1}$ is $\rho m^{-1}$- firmly nonexpansive.

### Proof

See Theorems 2.2.35 and 2.2.42 in [13] along with Theorem 2.12 above. $\square$

### Definition 2.18

An operator $T:\mathcal {H\rightarrow \mathcal {H}}$ is *weakly regular* (i.e., satisfies *Opial’s demi-closedness principle which says that* $T-Id$ *is demiclosed at 0*) if for any sequence $\left\{ x^{k}\right\} _{k=0}^{\infty }\subset \mathcal {H}$ and any $x\in \mathcal {H}$, the following implication holds:

$$ {\left\{ \begin{array}{ll} \begin{array}{c} x^{k}\rightharpoonup x\\ T\left( x^{k}\right) -x^{k}\rightarrow 0 \end{array}\Longrightarrow x\in \textrm{Fix}T \end{array}\right. } $$

### Lemma 2.19

Let $T:\mathcal {H}\rightarrow \mathcal {H}$ be nonexpansive. Then *T* is weakly regular.

### Proof

See Lemma 3.2.5 in [13]. $\square$

### Example 2.20

Given a nonempty, closed an convex subset *C* of $\mathcal {H}$, the metric projection $P_{C}$ onto *C* is firmly nonexpansive (see, e.g., Theorem 2.2.21 in [13]) and, hence, a nonexpansive cutter (by Theorem 2.10) and weakly regular (by Lemma 2.19). Moreover $\textrm{Fix}P_{C}=C$.

### Definition 2.21

For a nonempty, closed and convex subset *C* of $\mathcal {H}$, the sequence $\left\{ x^{k}\right\} _{k=0}^{\infty }$ in $\mathcal {H}$ is

(i) *Fejér monotone* w*ith respect to C* if for each $z\in C$ and each $k\in \mathbb {N}$,
  $$ \left\| x^{k+1}-z\right\| \le \left\| x^{k}-z\right\| . $$
(ii) *Strictly Fejér monotone* *with respect to C* if for each $z\in C$ and each $k\in \mathbb {N}$,
  $$ \left\| x^{k+1}-z\right\| <\left\| x^{k}-z\right\| . $$

### Theorem 2.22

Let *C* be a nonempty, closed and convex subset of $\mathcal {H}$. If $\left\{ x^{k}\right\} _{k=0}^{\infty }$ is Fejér monotone with respect to *C*, then it converges strongly to some point in *C* if and only if

$$ \lim _{k\rightarrow \infty }d\left( x^{k},C\right) =0. $$

### Proof

See Theorems 2.16(v) in [8]. $\square$

Next we investigate the properties of the following algorithm which is the algorithmic framework for our GDSA method.

### Algorithm 2.23

(The algorithmic framework) Given $\varepsilon \in \left( 0,1\right]$, $x^{0}\in \mathcal {H}$ and a sequence $\left\{ T_{k}\right\} _{k=0}^{\infty }$ of operators,$T_{k}:\mathcal {H\rightarrow \mathcal {H}}$ for each $k\in \mathbb {N}$, let the algorithm be defined by the recurrence

$$ x^{k+1}:=x^{k}+\lambda _{k}\left( T_{k}\left( x^{k}\right) -x^{k}\right) , $$

where $\lambda _{k}\in \left[ \varepsilon ,2-\varepsilon \right]$ for each $k\in \mathbb {N}$.

The next definition of bounded perturbations resilience of an iterative algorithm that is governed by an infinite sequence of algorithmic operators generalizes the earlier commonly used definition, e.g., Definition 3.1 in [22], wherein only a single operator was considered. See [11], where it was shown for the first time that if the exact iterates of a nonexpansive operator converge, then its inexact iterates with summable errors converge as well.

### Definition 2.24

**Bounded perturbations resilience**. Let $\Gamma \subseteq \mathcal {H}$ be a given nonempty subset of $\mathcal {H}$ and $\left\{ T_{k}\right\} _{k=0}^{\infty }$ be a sequence of operators, $T_{k}:\mathcal {H}\rightarrow \mathcal {H}$ for each $k\in \mathbb {N}$. The algorithm $x^{k+1}:=T_{k}(x^{k}),$ for all $k\in \mathbb {N},$ is said to be *weakly (strongly) bounded perturbations resilient* with respect to $\Gamma$ if the following is true: If a sequence $\{x^{k}\}_{k=0}^{\infty },$ generated by the algorithm, converges weakly (strongly) to a point in $\Gamma$ for all $x^{0}\in \mathcal {H}$, then any sequence $\{y^{k}\}_{k=0}^{\infty }$ in $\mathcal {H}$ that is generated by the algorithm $y^{k+1}:=T_{k}(y^{k}+\beta _{k}v^{k}),$ for all $k\in \mathbb {N},$ also converges weakly (strongly) to a point in $\Gamma$ for all $y^{0}\in \mathcal {H},$ provided that $\{\beta _{k}v^{k}\}_{k=0}^{\infty }$ are bounded perturbations, meaning that $\left\{ \beta _{k}\right\} _{k=0}^{\infty }$ is a sequence of positive real numbers such that $\sum _{k=0}^{\infty }\beta _{k}<\infty$ and that the sequence $\{v^{k}\}_{k=0}^{\infty }$ is a bounded sequence in $\mathcal {H}$.

### Remark 2.25

The terms *“weakly (strongly)”* in the above definition are related to the convergence of the considered sequences. They should not be confused with the notions of weak and strong perturbation resilience used in the theory of the superiorization method, see [17], particularly Definition 9 therein.

The notions of coherence and strong coherence presented next play a fundamental role in our work here.

### Definition 2.26

A sequence $\left\{ T_{k}\right\} _{k=0}^{\infty }$ of self-mapping operators of $\mathcal {H}$ with the set of common fixed points $F:=\cap _{k=0}^{\infty }\textrm{Fix}T_{k}$ is:

(i) *Coherent* if for every bounded sequence $\left\{ z^{k}\right\} _{k=0}^{\infty }$ in $\mathcal {H}$, we have
  $$ {\left\{ \begin{array}{ll} \begin{array}{c} \sum _{k=0}^{\infty }\Vert z^{k+1}-z^{k}\Vert ^{2}<\infty \\ \sum _{k=0}^{\infty }\Vert T_{k}\left( z^{k}\right) -z^{k}\Vert ^{2}<\infty \end{array}\Longrightarrow \mathfrak {W}\left( \left\{ z^{k} \right\} _{k=0}^{\infty }\right) \subset F. \end{array}\right. } $$
(ii) *Strongly coherent* if for any bounded sequence $\left\{ z^{k}\right\} _{k=0}^{\infty }$ in $\mathcal {H}$, we have
  $$ {\left\{ \begin{array}{ll} \begin{array}{c} z^{k+1}-z^{k}\rightarrow 0\\ T_{k}\left( z^{k}\right) -z^{k}\rightarrow 0 \end{array}\Longrightarrow \mathfrak {W}\left( \left\{ z^{k} \right\} _{k=0}^{\infty }\right) \subset F. \end{array}\right. } $$

### Remark 2.27

Clearly, a strongly coherent sequence of operators is coherent. See Example 3.3 in [5] to verify that this inclusion is proper.

### Lemma 2.28

Let $\delta \in \left( 0,1\right]$ be a fixed real number, $\left\{ \lambda _{k}\right\} _{k=0}^{\infty }$ be a real sequence such that $\lambda _{k}\in \left[ \delta ,1\right]$ for each $k\in \mathbb {N}$ and let $\left\{ T_{k}\right\} _{k=0}^{\infty }$ be a sequence of operators, $T_{k}:\mathcal {H}\rightarrow \mathcal {H}$ for each $k\in \mathbb {N}$. Then the sequence $\left\{ T_{k\lambda _{k}}\right\} _{k=0}^{\infty }$ of $\lambda _{k}$-relaxations of $\left\{ T_{k}\right\} _{k=0}^{\infty }$, that is, $T_{k\lambda _{k}}:=Id+\lambda _{k}\left( T_{k}-Id\right)$ for each $k\in \mathbb {N}$, is coherent if and only if $\left\{ T_{k}\right\} _{k=0}^{\infty }$ is coherent.

### Proof

See Proposition 4.5 in [9]. $\square$

### Remark 2.29

Algorithm 2.23 has a slightly different formulation in [9], where $\lambda _{k}:=2-\varepsilon$ for each $k\in \mathbb {N}$. Due to Remark 2.7 and Lemma 2.28, the following theorem (presented in [9]) is valid in the case of our adjusted algorithm above as well.

### Theorem 2.30

Suppose that $\left\{ T_{k}\right\} _{k=0}^{\infty }$ is a coherent sequence of cutters. If $\cap _{k=0}^{\infty }\textrm{Fix}T_{k}\not =\emptyset$, then the sequence defined by Algorithm 2.23 converges weakly to some $x\in \cap _{k=0}^{\infty }\textrm{Fix}T_{k}$.

### Proof

See Theorem 4.2*(i)* in [9]. $\square$

The following theorem is a slight extension of Theorem 3.13 in [5]. We prove it here in the case of strong coherence for the convenience of the reader.

### Theorem 2.31

Let *I* be an arbitrary nonempty index set and let $\left\{ C_{i}\right\} _{i\in I}$ be a family of (possibly empty) closed and convex sets in $\mathcal {H}$ and let $\left\{ T_{k}\right\} _{k=0}^{\infty }$ be a sequence of operators, $S_{k}:\mathcal {H\rightarrow \mathcal {H}}$ for each $k\in \mathbb {N}$. Assume that

2.3 $$\begin{aligned} C:=\cap _{i\in I}C_{i}\subset F:=\cap _{k=0}^{\infty }\textrm{Fix}T_{k} \end{aligned}$$

and $\left\{ I_{k}\right\} _{k=0}^{\infty }$ is an admissible control sequence of subsets of $\mathbb {N}$, that is, for each $i\in I$, there is an integer $M_{i}>0$ such that

2.4 $$\begin{aligned} i\in \cup _{n=k}^{k+M_{i}-1}I_{n}\,\,\mathrm {for\,all}\,k\in \mathbb {N}. \end{aligned}$$

Finally, suppose that for every $i\in I$, every $z\in \mathcal {H}$, every bounded sequence $\left\{ z^{k}\right\} _{k=0}^{\infty }$ in ${\mathcal {H}}$, and every strictly increasing sequence $\left\{ n_{k}\right\} _{k=0}^{\infty }\subset \mathbb {N}$, we have

2.5 $$\begin{aligned} {\left\{ \begin{array}{ll} z^{n_{k}}\rightharpoonup z\\ i\in I_{n_{k}}\,\,\mathrm {for\,all}\,k\in \mathbb {N}\\ z^{k+1}-z^{k}\rightarrow 0\\ T_{k}\left( z^{k}\right) -z^{k}\rightarrow 0 \end{array}\right. }\Longrightarrow z\in C_{i}. \end{aligned}$$

Then the sequence $\left\{ T_{k}\right\} _{k=0}^{\infty }$ is strongly coherent.

### Proof

We first show that under the assumptions of the theorem we have for each bounded sequence $\left\{ z^{k}\right\} _{k=0}^{\infty }$ in $\mathcal {H}$,

2.6 $$\begin{aligned} {\left\{ \begin{array}{ll} z^{k+1}-z^{k}\rightarrow 0\\ T_{k}\left( z^{k}\right) -z^{k}\rightarrow 0 \end{array}\right. }\Longrightarrow \mathfrak {W}\left( \left\{ z^{k} \right\} _{k=0}^{\infty }\right) \subset C. \end{aligned}$$

Let $\left\{ z^{k}\right\} _{k=0}^{\infty }$ be a bounded sequence in $\mathcal {H}$ satisfying

2.7 $$\begin{aligned} {\left\{ \begin{array}{ll} z^{k+1}-z^{k}\rightarrow 0\\ T_{k}\left( z^{k}\right) -z^{k}\rightarrow 0 \end{array}\right. } \end{aligned}$$

and let $z\in \mathfrak {W}\left( \left\{ z^{k} \right\} _{k=0}^{\infty }\right)$. Then there is a strictly increasing sequence $\left\{ n_{k}\right\} _{k=0}^{\infty }$ of natural numbers such that $z^{n_{k}}\rightharpoonup z$. Suppose that $i\in I$. By (2.4), there is an $M_{i}$ such that the condition in (2.4) holds for all $k\in \mathbb {N}$. Therefore, there is a sequence $\left\{ p_{k}\right\} _{k=0}^{\infty }$ in $\mathbb {N}$ such that

$$ \left( \forall k\in \mathbb {N}\right) n_{k}\le p_{k}\le n_{k}+M_{i}-1\,\,\,\textrm{and}\,\,\,i\in I_{p_{k}}. $$

Without any loss of generality, we may assume that $n_{k+1}-n_{k}\ge M_{i}$ for each $k\in \mathbb {N}$, because otherwise we can choose a subsequence of $\left\{ z^{n_{k}}\right\} _{k=0}^{\infty }$ with this property. Thus, we can assume that $\left\{ p_{k}\right\} _{k=0}^{\infty }$ is strictly increasing. Moreover,

2.8 $$\begin{aligned} \left( \forall k\in \mathbb {N}\right) z^{p_{k}}=\sum _{j=0}^{p_{k}-1-n_{k}}\left( z^{n_{k}+j+1}-z^{n_{k}+j}\right) +z^{n_{k}}. \end{aligned}$$

(By definition $\sum _{j=0}^{-1}\left( z^{n_{k}+j+1}-z^{n_{k}+j}\right) :=0$). By (2.7) and due to the finite number of summands in (2.8), which is at most $M_{i}$, we obtain $z^{p_{k}}\rightharpoonup z$. By the condition in (2.5) with respect to the sequence $\left\{ p_{k}\right\} _{k=0}^{\infty }$, we have $z\in C_{i}$. This is true for each $i\in I$; hence, $z\in C$. Thus, $\mathfrak {W}\left( \left\{ z^{k} \right\} _{k=0}^{\infty }\right) \subset C$ and (2.6) holds. Combining this with (2.3), we see that the sequence $\left\{ T_{k}\right\} _{k=0}^{\infty }$ is strongly coherent. Theorem 2.31 is now proved. $\square$

### Remark 2.32

Under the assumptions of Theorem 2.31, if all $\left\{ T_{k}\right\} _{k=0}^{\infty }$ are cutters then Theorem 2.30 holds true when applied to $\left\{ T_{k}\right\} _{k=0}^{\infty }$ and *F* is replaced by *C*. See Theorem 2.2.1 and Remark 2.2.4 in [3] in this connection.

### Theorem 2.33

Let $C\subset \mathcal {H}$ be nonempty and closed. Let $\left\{ T_{k}\right\} _{k=0}^{\infty }$, $T_{k}:{\mathcal {H}\rightarrow \mathcal {H}}$ for each $k\in \mathbb {N}$, be a sequence of nonexpansive operators satisfying $C\subset \cap _{k=0}^{\infty }\textrm{Fix}T_{k}$. Assume that for each $y\in \mathcal {H}$ and each $q\in \mathbb {N}$, the sequence $\left\{ T_{q+k}\cdots T_{q+1}T_{q}\left( y\right) \right\} _{k=0}^{\infty }$ converges weakly (strongly) to an element of *C*. Let $\left\{ \gamma _{k}\right\} _{k=0}^{\infty }\subset \left[ 0,\infty \right)$ be a sequence such that $\sum _{\text {k}=0}^{\infty }\gamma _{k}<\infty$ and let $\left\{ y^{k}\right\} _{k=0}^{\infty }\subset \mathcal {H}$. Further assume that for each $k\in \mathbb {N}$,

$$ \left\| y^{k+1}-T_{k}\left( y^{k}\right) \right\| \le \gamma _{k}. $$

Then the sequence $\left\{ y^{k}\right\} _{k=0}^{\infty }$converges weakly (strongly) to an element of *C*.

### Proof

See Theorems 3.2 and 5.2 in [12]. $\square$

In the sequel we employ the following useful property of a convex and continuous function.

### Theorem 2.34

Let the function $\phi :\mathcal {H}\rightarrow \mathbb {R}$ be convex and continuous at the point $x\in \mathcal {H}$. Then the subgradient set $\partial \phi \left( x\right)$ is nonempty.

### Proof

See Theorem 16.17(ii) in [10]. $\square$

## The General Bounded Regularity and Approximately Shrinking Operators

In this section we consider the notions of bounded regularity and approximate shrinking which are needed for establishing the strong convergence of our algorithms.

The property of bounded regularity of a finite family of sets was studied in [8, Section 5] and [7]. Below we expand its definition to hold for an arbitrary family of sets and show that such a general boundedly regular family of sets possesses the same properties as a finite one.

### Definition 3.1

For a nonempty index set *I*, the family $\left\{ C_{i}\right\} _{i\in I}$ of nonempty, closed and convex subsets of $\mathcal {H}$ with nonempty intersection *C* is *boundedly regular *if for any bounded sequence $\left\{ x^{k}\right\} _{k=0}^{\infty }$ in $\mathcal {H}$, the following implication holds:

$$ \lim _{k\rightarrow \infty }d\left( x^{k},C_{i}\right) =0\,\,\textrm{for}\,\,\mathrm {each\,\,}i\in I\,\,\Longrightarrow \,\,\lim _{k\rightarrow \infty }d\left( x^{k},C\right) =0. $$

The next proposition provides sufficient conditions for bounded regularity. We recall that a topological space *X* is locally compact if each $x\in X$ has a compact neighborhood with respect to the topology inherited from *X*.

### Proposition 3.2

Let $\left\{ C_{i}\right\} _{i\in I}$ be a family of nonempty, closed and convex subsets of $\mathcal {H}$ with a nonempty intersection *C*. Then the following assertions hold:

(i) If there is $i_{0}\in I$ for which the set $C_{i_{0}}$ is a locally compact topological space (with respect to the norm topology inherited from $\mathcal {H}$), then the family $\left\{ C_{i}\right\} _{i\in I}$ is boundedly regular.
(ii) If $\mathcal {H}$ is of finite dimension, then the family $\left\{ C_{i}\right\} _{i\in I}$ is boundedly regular.

### Proof

*(i)* Let $i_{0}\in I$ be an index for which the set $C_{i_{0}}$ is a locally compact topological space with respect to the norm topology inherited from $\mathcal {H}$. Let $\left\{ x^{k}\right\} _{k=0}^{\infty }\subset \mathcal {H}$ be a bounded sequence such that

3.1 $$\begin{aligned} \lim _{k\rightarrow \infty }d\left( x^{k},C_{i}\right) =0\,\,\textrm{for}\,\,\mathrm {each\,\,}i\in I. \end{aligned}$$

We need to show that $\lim _{k\rightarrow \infty }d\left( x^{k},C\right) =0.$ Assume to the contrary that this is not true. Then, since the sequence $\left\{ x^{k}\right\} _{k=0}^{\infty }$ is bounded, we may assume, without any loss of generality, that $\lim _{k\rightarrow \infty }d\left( x^{k},C\right)$ exists in $\mathbb {R}$,

3.2 $$\begin{aligned} \lim _{k\rightarrow \infty }d\left( x^{k},C\right) \not =0 \end{aligned}$$

and $\left\{ x^{k}\right\} _{k=0}^{\infty }$ converges weakly to some point $x\in \mathcal {H}$, because otherwise there exists a subsequence $\left\{ x^{n_{k}}\right\} _{k=0}^{\infty }$ of $\left\{ x^{k}\right\} _{k=0}^{\infty }$ with these properties. By (3.1), we see that

3.3 $$\begin{aligned} x^{k}-P_{C_{i_{0}}}\left( x^{k}\right) \rightarrow 0. \end{aligned}$$

It follows from (3.3) that the sequence $\left\{ P_{C_{i_{0}}}\left( x^{k}\right) \right\} _{k=0}^{\infty }\subset C_{i_{0}}$ also converges weakly to *x* and hence is bounded. Since $C_{i_{0}}$ is closed and convex, its local compactness implies its bounded compactness (that is, each closed ball in $C_{i_{0}}$, with respect to the norm topology inherited from $\mathcal {H}$, is compact). Therefore, the bounded sequence $\left\{ P_{C_{i_{0}}}\left( x^{k}\right) \right\} _{k=0}^{\infty }$ has a subsequence which converges strongly to *x*. Thus, we may assume that $\left\{ P_{C_{i_{0}}}\left( x^{k}\right) \right\} _{k=0}^{\infty }$ converges strongly to *x*. By (3.3), the sequence $\left\{ x^{k}\right\} _{k=0}^{\infty }$ also converges strongly to *x*. Now let $i\in I$ and let $\varepsilon >0$ be an arbitrary positive number. Then there exists a $k_{0}\in \mathbb {N}$ such that $\left\| x^{k}-x\right\| <\varepsilon$ for each natural $k\ge k_{0}$, and hence, for each $c\in C_{i}$, we obtain, by the triangle inequality,

3.4 $$\begin{aligned} d\left( x,C_{i}\right) \le \left\| x-c\right\| \le \left\| x-x^{k}\right\| +\left\| x^{k}-c\right\| <\varepsilon +\left\| x^{k}-c\right\| , \end{aligned}$$

for each natural $k\ge k_{0}$. Since (3.4) holds for an arbitrary $c\in C_{i}$, we obtain, by (3.1), that for each natural $k\ge k_{0}$,

3.5 $$\begin{aligned} d\left( x,C_{i}\right) <\varepsilon +d\left( x^{k},C_{i}\right) \rightarrow \varepsilon . \end{aligned}$$

The arbitrariness of $\varepsilon$ along with (3.5) imply that $d\left( x,C_{i}\right) =0$ and since the set $C_{i}$ is closed, we have $x\in C_{i}$. It follows that $x\in C$ because $i\in I$ is arbitrary. By the continuity of the distance function,

$$ \lim _{k\rightarrow \infty }d\left( x^{k},C\right) =d\left( x,C\right) =0 $$

which contradicts (3.2).

*(ii)* Since any closed subset of a finite-dimensional space is locally compact, the result follows from *(i)*.

This completes the proof of Proposition 3.2. $\square$

### Remark 3.3

Note that the local compactness of the set $C_{i_{0}}$ in Proposition 3.2*(i)* can be replaced by the bounded compactness, since these properties are equivalent for a closed and convex subset of a normed linear space.

In the next example we show that the above conditions are sufficient, but not necessary, for bounded regularity, as well as present an infinite family of nonempty, closed and convex subsets which is not boundedly regular. For an example of such a finite family, see, for instance, Example 5.5 in [7].

### Example 3.4

Set $\mathcal {H}:=l_{2}$. Let $\left\{ e^{k}\right\} _{k=0}^{\infty }$ be the sequence of elements in $l_{2}$, defined by $$e_{n}^{k}:={\left\{ \begin{array}{ll} 1, &  \textrm{if}\,\,n=k,\\ 0, &  \textrm{otherwise,} \end{array}\right. }$$ for each $n\in \mathbb {N}$. Let $C_{n}:=\overline{\textrm{sp}\left\{ e^{k}\right\} _{k=n}^{\infty }}$ for each $n\in \mathbb {N}$, where $\textrm{sp}$ denotes the span of a set of vectors and the upper bar denotes the closure. Clearly, $\cap _{n\in \mathbb {N}}C_{n}:$ $=\left\{ 0\right\} \not =\emptyset$ and for each $n\in \mathbb {N}$, we have $e^{k}\in C_{n}$ for each natural $k\ge n$. This implies that $\lim _{k\rightarrow \infty }d\left( e^{k},C_{i}\right) =0$ for each $n\in \mathbb {N}$. However, $\lim _{k\rightarrow \infty }d\left( e^{k},C\right) =1\not =0$ and, therefore, the family $\left\{ C_{n}\right\} _{n\in \mathbb {N}}$ is not boundedly regular.

Observe that if we choose $C_{n}:=\textrm{sp}\left\{ e^{n}\right\}$ for each $n\in \mathbb {\mathbb {N}}$ in the setting of this example, then by Proposition 3.2, the family $\left\{ C_{n}\right\} _{n\in \mathbb {N}}$ is a boundedly regular family of nonempty, closed and convex subsets of $\mathcal {H}$ with the nonempty intersection $\cap _{n\in \mathbb {N}}C_{n}:$ $=\left\{ 0\right\} \not =\emptyset$, since each $C_{n}$ is a finite-dimensional normed linear space and hence is locally compact.

Note that the local compactness in Proposition 3.2 is a sufficient condition for the bounded regularity, but not a necessary one. For instance, if we choose in the settings of this example $C_{n}:=\overline{B\left( 0,\left( n+1\right) ^{-1}\right) }$ for each $n\in \mathbb {N}$, then $\left\{ C_{n}\right\} _{n\in \mathbb {N}}$ is a regularly bounded family of nonempty closed and convex subsets of $\mathcal {H}$ with the nonempty intersection $\cap _{n\in \mathbb {N}}C_{n}=\left\{ 0\right\}$, but for each $n\in \mathbb {N}$, $C_{n}$ is not a locally compact subspace of $\mathcal {H}$.

We also recall the following notion of approximate shrinking which was extensively studied in [15].

### Definition 3.5

A quasi-nonexpansive operator $T:\mathcal {H}\rightarrow \mathcal {H}$ *is approximately shrinking* if for each bounded sequence $\left\{ x^{k}\right\} _{k=0}^{\infty }$ in $\mathcal {H}$, the following implication holds:

$$ \lim _{k\rightarrow \infty }\left\| T\left( x^{k}\right) -x^{k}\right\| =0\,\,\Longrightarrow \,\,\lim _{k\rightarrow \infty }d\left( x^{k},\textrm{Fix}T\right) =0. $$

### Example 3.6

Given a nonempty, closed and convex subset *C* of $\mathcal {H}$, the metric projection $P_{C}$ onto *C* is approximately shrinking (see Example 3.5 in [15]). However, the quasi-nonexpansive operator $U:\mathcal {H\rightarrow \mathcal {H}}$ defined by $$U\left( x\right) :={\left\{ \begin{array}{ll} P_{\overline{B\left( 0,2\right) }}\left( x\right) , &  \textrm{if}\,\,x\not \in \overline{B\left( 0,2\right) ,}\\ P_{\overline{B\left( 0,1\right) }}\left( x\right) , &  \textrm{if}\,\,x\in \overline{B\left( 0,2\right) ,} \end{array}\right. }$$ for each $x\in \mathcal {H}$ is not an approximately shrinking one (see Example 3.7 on p. 404 in [15]).

## The Convergence and Bounded Perturbation Resilience of the GDSA Method

In this section we consider a sequence $\left\{ T_{\left( \varOmega _{k},\omega _{k}\right) }\right\} _{k=0}^{\infty }$, defined below, of nonexpansive operators, under the assumption of an admissible control, with respect to which Algorithm 2.23 converges to a point in a certain set and is bounded perturbations resilient.

### Notions, Notations and Initial Tools

Throughout the rest of the paper we refer, among other things, to the following setting defined below. We recall that for an arbitrary sequence of sets $\left\{ A_{k}\right\} _{k=0}^{\infty }$, $\limsup _{k\rightarrow \infty }A_{k}:=\cap _{n=0}^{\infty }\cup _{k=n}^{\infty }A_{k}$.

Let *m* be a positive integer. We consider a finite family $\left\{ U_{i}\right\} _{i=1}^{m}$ of $\alpha _{i}$-relaxed firmly nonexpansive operators, where $U_{i}:\mathcal {H\rightarrow \mathcal {H}}$ and $\alpha _{i}\in \left( 0,2\right]$ for each $i=1,2,\dots ,m$. By Theorem 2.11, the operator $U_{i}$ is nonexpansive for each $i=1,2,\dots ,m$. Set $\mathcal {M}:=\max _{k\in \mathbb {N}}q_{k}$ and

$$ \rho _{\left\{ U_{i}\right\} _{i=1}^{m}}:=\min \left\{ \mathcal {M}^{-1}\min _{i\in \left\{ 1,2,\dots ,m\right\} }\left( 2-\alpha _{i}\right) \alpha _{i}^{-1},1\right\} \le 1. $$

Let $\left\{ q_{k}\right\} _{k=0}^{\infty }$ be a bounded sequence of positive integers, $\left\{ \varOmega _{k}\right\} _{k=0}^{\infty }$ be a family of nonempty sets such that $\varOmega _{k}\subset \left\{ 1,2,\dots ,m\right\} {EMPTY}^{\left\{ 1,2,\dots ,q_{k}\right\} }.$ That is, $\varOmega _{k}$ is a finite subset of the set of functions from $\left\{ 1,2,\dots ,q_{k}\right\}$ to $\left\{ 1,2,\dots ,m\right\}$ for each $k\in \mathbb {N}$. Since the sequence $\left\{ q_{k}\right\} _{k=0}^{\infty }$ is bounded, the number of different elements in the family $\left\{ \varOmega _{k}\right\} _{k=0}^{\infty }$ is finite. For each $k\in \mathbb {N}$ and each $t\in \varOmega _{k}$, set $V_{k}\left[ t\right] :=U_{t\left( q_{k}\right) }\cdots U_{t\left( 2\right) }U_{t\left( 1\right) }$ and let $\omega _{k}:\varOmega _{k}\rightarrow \left( 0,1\right]$ be a function such that $\sum _{t\in \varOmega _{k}}\omega _{k}\left( t\right) =1$. For each $k\in \mathbb {N}$, define $T_{\left( \varOmega _{k},\omega _{k}\right) }:=\sum _{t\in \varOmega _{k}}\omega _{k}\left( t\right) V_{k}\left[ t\right]$. Define the set $I:=\limsup _{k\rightarrow \infty }\left\{ T_{\left( \varOmega _{k},\omega _{k}\right) }\right\}$, that is, $I=\limsup _{k\rightarrow \infty }A_{k}$, where the sequence of sets $\left\{ A_{k}\right\} _{k=0}^{\infty }$ is defined by singletones, $A_{k}:=\left\{ T_{\left( \varOmega _{k},\omega _{k}\right) }\right\}$. Define a family of sets $\left\{ C_{T}\right\} _{T\in I}$ by $C_{T}:=\textrm{Fix}T$ for each $T\in I$. Set $F:=\cap _{k=0}^{\infty }\textrm{Fix}T_{\left( \varOmega _{k},\omega _{k}\right) }$ and $C:=\cap _{T\in I}C_{T}$. For each $k\in \mathbb {N}$, we say that a set $\varOmega _{k}$ is *fit* if

$$ \cup _{t\in \varOmega _{k}}{{\,\textrm{Im}\,}}t=\left\{ 1,2,\dots ,m\right\} , $$

where ${{\,\textrm{Im}\,}}t$ denotes the image of the mapping *t*.

#### Remark 4.1

The operators $T_{\left( \varOmega _{k},\omega _{k}\right) }$, defined above, are “string-averaging operators” as first introduced in [19] and further studied in various forms and settings, see, for instance, Example 5.21 in [10, 27] and [28], to name but a few. In those and other papers, the index vector *t* is called “a string”, the composite operator $V_{k}\left[ t\right]$ is called “a string operator” and $\omega _{k}$ are called “weight functions”.

We introduce the following definition of $\limsup$-admissibility of sequences of operators.

#### Definition 4.2

We say that a sequence $\left\{ T_{k}\right\} _{k=0}^{\infty }$of operators, $T_{k}:\mathcal {H}\rightarrow \mathcal {H}$ for each $k\in \mathbb {N}$, is $\limsup$*-admissible*, if $\left\{ T_{k}\right\} _{k=0}^{\infty }\subset \limsup _{k\rightarrow \infty }\left\{ T_{k}\right\}$ and for each $T\in \limsup _{k\rightarrow \infty }\left\{ T_{k}\right\}$, there is an integer $M_{T}>0$ such that $T\in \cup _{n=k}^{k+M_{T}-1}\left\{ T_{\left( \varOmega _{n},w_{n}\right) }\right\}$ for all $k\in \mathbb {N}$.

#### Remark 4.3

Clearly, for each $k_{0}\in \mathbb {N}$, $\limsup _{k\rightarrow \infty }\left\{ T_{k}\right\} =\limsup _{k\rightarrow \infty }\left\{ T_{k_{0}+k}\right\}$ and if a sequence $\left\{ T_{k}\right\} _{k=0}^{\infty }$ of operators is $\limsup$-admissible, then the sequence $\left\{ T_{k_{0}+k}\right\} _{k=0}^{\infty }$ is $\limsup$-admissible. We use this observation in the sequel.

#### Remark 4.4

Observe that if, in the above setting, we require the sequence $\left\{ \omega _{k}\right\} _{k=0}^{\infty }$ to attain a finite number of values, then the sequence $\left\{ T_{\left( \varOmega _{k},\omega _{k}\right) }\right\} _{k=0}^{\infty }$ will also attain a finite number of values. Thus, in order to ensure the existence of $k_{0}\in \mathbb {N}$ such that the sequence $\left\{ T_{\left( \varOmega _{k_{0}+k},\omega _{k_{0}+k}\right) }\right\} _{k=0}^{\infty }$ is $\limsup$-admissible, we only need to require the existence of an integer $M_{T}>0$, for each $T\in I$, such that $T\in \cup _{n=k}^{n+M_{T}-1}\left\{ T_{\left( \varOmega _{n},w_{n}\right) }\right\}$ for all $k\in \mathbb {N}$.

### The Convergent and Bounded Perturbation Resilient GDSA Method

In this subsection we present several lemmata leading to our main result (Theorem 4.8 below) that gives conditions under which the GDSA method converges and is bounded perturbations resilient. All notions and notations are those presented in Subsection 4.1 above.

We consider the following algorithm which is actually Algorithm 2.23 with respect to the operators $\left\{ T_{\left( \varOmega _{k},\omega _{k}\right) }\right\} _{k=0}^{\infty }$ which were defined in the settings of the previous subsection.

#### Algorithm 4.5

(The General Dynamic String-Averaging (GDSA) algorithm) Given $\varepsilon \in \left( 0,1\right]$, $x^{0}\in \mathcal {H}$ and a sequence $\left\{ T_{\left( \varOmega _{k},\omega _{k}\right) }\right\} _{k=0}^{\infty }$ of operators, the algorithm is defined by the recurrence

$$ x^{k+1}:=x^{k}+\lambda _{k}\left( T_{\left( \varOmega _{k},\omega _{k}\right) }\left( x^{k}\right) -x^{k}\right) , $$

where $\lambda _{k}\in \left[ \varepsilon ,1+\rho _{\left\{ U_{i}\right\} _{i=1}^{m}}-\varepsilon \right]$ for each $k\in \mathbb {N}$.

Next we show that under $\limsup$-admissibility Algorithm 4.5 is strongly coherent.

#### Lemma 4.6

Assume that $\left\{ T_{\left( \varOmega _{k},\omega _{k}\right) }\right\} _{k=0}^{\infty }$ is $\limsup$-admissible and let $x_{0}\in \mathcal {H}$. Then $C=F$ and the sequence generated by Algorithm 4.5 is strongly coherent and hence coherent.

#### Proof

Definitely, $C=F$. Define a sequence $\left\{ I_{k}\right\} _{k=0}^{\infty }$ of singletons of the elements from the set *I* by $I_{k}:=\left\{ T_{\left( \varOmega _{k},\omega _{k}\right) }\right\}$ for each $k\in \mathbb {N}$. Since for each $k\in \mathbb {N}$ and each $T\in I$, we have $T=T_{\left( \varOmega _{m},\omega _{m}\right) }$ for some $m\in \left\{ k,k+1,\dots ,k+M_{T}-1\right\}$, it follows that each $T\in I$ satisfies $T\in \cup {EMPTY}_{n=k}^{k+M_{T}-1}I_{n}$ for all $k\in \mathbb {N}$. Now assume that $T\in I$, let $z\in \mathcal {H}$, let $\left\{ z^{k}\right\} _{k=0}^{\infty }$ be a bounded sequence in $\mathcal {H}$, satisfying

4.1 $$\begin{aligned} z^{k+1}-z^{k}\rightarrow 0\,\,\mathrm {and\,\,}T_{\left( \varOmega _{k},\omega _{k}\right) }\left( z^{k}\right) -z^{k}\rightarrow 0, \end{aligned}$$

and let $\left\{ n_{k}\right\} _{k=0}^{\infty }$ be a strictly increasing sequence of natural numbers such that $z^{n_{k}}\rightharpoonup z\in \mathcal {H}$ and $T\in I_{n_{k}}$ for all $k\in \mathbb {N}$. Clearly, $T_{\left( \varOmega _{n_{k}},\omega _{n_{k}}\right) }=T$ for all $k\in \mathbb {N}$. By (4.1), we have ${T}\mathinner {\left( z^{n_{k}}\right) }-z^{n_{k}}\rightarrow 0$ and since $z^{n_{k}}\rightharpoonup z$, it follows from the weak regularity of the operator *T* (see Theorem 2.10, Theorem 2.11, Remark 2.3 and Lemma 2.19 above) that $z\in \textrm{Fix}T=C_{T}$. This yields that the condition in (2.5) holds. Thus, the sequence $\left\{ T_{\left( \varOmega _{k},\omega _{k}\right) }\right\} _{k=0}^{\infty }$ is strongly coherent by Theorem 2.31 and hence coherent. This completes the proof of the lemma. $\square$

The next result tells about the $\lambda _{k}$-relaxation of $T_{\left( \varOmega _{k},\omega _{k}\right) }$.

#### Lemma 4.7

Let $\left\{ \lambda _{k}\right\} _{k=0}^{\infty }$ be a sequence of real numbers such that $\lambda _{k}\in \left[ \varepsilon ,1+\rho _{\left\{ U_{i}\right\} _{i=1}^{m}}-\varepsilon \right]$ for each $k\in \mathbb {N}$, where $\varepsilon >0$. Then there exists a sequence $\left\{ S_{k}\right\} _{k=0}^{\infty }$ of firmly nonexpansive operators, $S_{k}:\mathcal {H\rightarrow \mathcal {H}}$ for each $k\in \mathbb {N}$, such that $T_{\left( \varOmega _{k},\omega _{k}\right) \lambda _{k}}$, the $\lambda _{k}$-relaxation of $T_{\left( \varOmega _{k},\omega _{k}\right) }$, satisfies

4.2 $$\begin{aligned} T_{\left( \varOmega _{k},\omega _{k}\right) \lambda _{k}}=Id+2\lambda _{k}\left( 1+\rho _{\left\{ U_{i}\right\} _{i=1}^{m}}\right) ^{-1}\left( S_{k}-Id\right) , \end{aligned}$$

where

4.3 $$\begin{aligned} 2\lambda _{k}\left( 1+\rho _{\left\{ U_{i}\right\} _{i=1}^{m}}\right) ^{-1}\in \left[ 2\varepsilon \left( 1+\rho _{\left\{ U_{i}\right\} _{i=1}^{m}}\right) ^{-1},2-2\varepsilon \left( 1+\rho _{\left\{ U_{i}\right\} _{i=1}^{m}}\right) ^{-1}\right] . \end{aligned}$$

for each $k\in \mathbb {N}$. Consequently, the operator $T_{\left( \varOmega _{k},\omega _{k}\right) \lambda _{k}}$ is $\left( 1+\rho _{\left\{ U_{i}\right\} _{i=1}^{m}}-\lambda _{k}\right) \lambda _{k}^{-1}$-firmly nonexpansive and, in particular, nonexpansive for each $k\in \mathbb {N}$.

#### Proof

By the definition of $\left\{ \lambda _{k}\right\} _{k=0}^{\infty }$, (4.3) holds. Define a sequence $\left\{ S_{k}\right\} _{k=0}^{\infty }$ of operators by

4.4 $$\begin{aligned} S_{k}:=Id+2^{-1}\left( 1+\rho _{\left\{ U_{i}\right\} _{i=1}^{m}}\right) \left( T_{\left( \varOmega _{k},\omega _{k}\right) }-Id\right) \end{aligned}$$

for each $k\in \mathbb {N}$. Set $\rho :=\min _{i\in \left\{ 1,2,\dots ,m\right\} }\left( 2-\alpha _{i}\right) \alpha _{i}^{-1}$. By Theorem 2.12, $U_{i}$ is $\rho$-firmly nonexpansive for each $i=1,2,\dots ,m$. By Corollary 2.17*(ii)*, for each $k\in \mathbb {N}$, the operator $V_{k}\left( t\right)$ is $\rho _{\left\{ U_{i}\right\} _{i=1}^{m}}$-firmly nonexpansive for each $t\in \varOmega _{k}$. As a result, by Corollary 2.17*(i)*, $T_{\left( \varOmega _{k},\omega _{k}\right) }$ is $\rho _{\left\{ U_{i}\right\} _{i=1}^{m}}$-firmly nonexpansive for each $k\in \mathbb {N}$ and by Corollary 2.13 and by (4.4), $S_{k}$ is firmly nonexpansive for each $k\in \mathbb {\mathbb {N}}$. Now $T_{\left( \varOmega _{k},\omega _{k}\right) \lambda _{k}}$ satisfies

4.5 $$\begin{aligned} T_{\left( \varOmega _{k},\omega _{k}\right) \lambda _{k}}&=Id+\lambda _{k}\left( T_{\left( \varOmega _{k},\omega _{k}\right) }{-}Id\right) {=}Id{+}2\lambda _{k}\left( 1{+}\rho _{\left\{ U_{i}\right\} _{i=1}^{m}}\right) ^{-1}\left( S_{k}{-}Id\right) \end{aligned}$$

for each $k\in \mathbb {N}$, so (4.2) holds. Hence, by (4.5) and (4.3), $T_{\left( \varOmega _{k},\omega _{k}\right) \lambda _{k}}$ is a $2\lambda _{k}\left( 1+\rho _{\left\{ U_{i}\right\} _{i=1}^{m}}\right) ^{-1}$-relaxed firmly nonexpansive operator for each $k\in \mathbb {N}$. By Theorem  2.12, $T_{\left( \varOmega _{k},\omega _{k}\right) \lambda _{k}}$ is $\left( 1+\rho _{\left\{ U_{i}\right\} _{i=1}^{m}}-\lambda _{k}\right) \lambda _{k}^{-1}$-firmly nonexpansive and, in particular, nonexpansive for each $k\in \mathbb {N}$, and the lemma is proved. $\square$

The next theorem is the main result of this paper. It gives conditions under which our GDSA algorithm converges and is a bounded perturbations resilient method. Analogous results in a somewhat more general framework for the consistent case were presented in [29], where assumptions of a similar nature were made on the input operators of a certain procedure. This was possible due to the existence of a common fixed point of those input operators. Since in this work we consider the inconsistent case, we need to rely on the assumptions regarding the output operators of our GDSA procedure.

#### Theorem 4.8

Assume that $C\not =\emptyset$ and that the sequence $\left\{ T_{\left( \varOmega _{k},\omega _{k}\right) }\right\} _{k=0}^{\infty }$ is $\limsup$-admissible. Let $\left\{ \lambda _{k}\right\} _{k=0}^{\infty }$ be a sequence of real numbers such that $\lambda _{k}\in \left[ \varepsilon ,1+\rho _{\left\{ U_{i}\right\} _{i=1}^{m}}-\varepsilon \right]$ for each $k\in \mathbb {N}$, where $\varepsilon >0$, and let $x^{0}\in \mathcal {H}$. Suppose that $\left\{ x^{k}\right\} _{k=0}^{\infty }$ is a sequence generated by Algorithm 4.5 with respect to the sequence $\left\{ \lambda _{k}\right\} _{k=0}^{\infty }$. Then the following assertions hold:

(i) The sequence $\left\{ x^{k}\right\} _{k=0}^{\infty }$ converges weakly to a point $x\in C$.
(ii) The sequence$\left\{ x^{k}\right\} _{k=0}^{\infty }$ is Fejér monotone with respect to *C*, namely,
  $$ \left\| x^{k+1}-z\right\| ^{2}\le \left\| x^{k}-z\right\| ^{2}-\varepsilon \left( 1+\rho _{\left\{ U_{i}\right\} _{i=1}^{m}}-\varepsilon \right) ^{-1}\left\| \left( x^{k+1}-x^{k}\right) \right\| ^{2} $$
  for each $k\in \mathbb {N}$, and, as a result, $\left\| x^{k+1}-x^{k}\right\| \rightarrow 0$.
(iii) If each $T\in I$ is approximately shrinking and the family $\left\{ C_{T}\right\} _{T\in I}$ is boundedly regular, then $\left\{ x^{k}\right\} _{k=0}^{\infty }$ converges strongly to a point $x\in C$.
(iv) The sequence$\left\{ x^{k}\right\} _{k=0}^{\infty }$ is weakly (strongly, if the convergence in (i) is strong) bounded perturbations resilient with respect to *C*.

#### Proof

*(i)* By Lemma 4.6, $F=C\not =\emptyset$ and the sequence $\left\{ T_{\left( \varOmega _{k},\omega _{k}\right) }\right\} _{k=0}^{\infty }$ is strongly coherent. By Lemma 4.7, there exists a sequence $\left\{ S_{k}\right\} _{k=0}^{\infty }$ of firmly nonexpansive operators, $S_{k}:\mathcal {H\rightarrow \mathcal {H}}$ for each $k\in \mathbb {N}$, such that (4.2) and (4.3) hold for each $k\in \mathbb {N}$. Hence the sequence $\left\{ S_{k}\right\} _{k=0}^{\infty }$ is also strongly coherent. Now we apply (4.2), (4.3), Remark 2.7, Theorem 2.10 and Theorem 2.30 to deduce that the sequence $\left\{ x^{k}\right\} _{k=0}^{\infty }$, generated by Algorithm 4.5, with respect to the sequence $\left\{ \lambda _{k}\right\} _{k=0}^{\infty }\subset \left[ \varepsilon ,1+\rho _{\left\{ U_{i}\right\} _{i=1}^{m}}-\varepsilon \right]$ converges weakly to a point $x\in C$.

*(ii)* Let $k\in \mathbb {N}$ be a natural number and $z\in C$. By the definition of $\left\{ x^{k}\right\} _{k=0}^{\infty }$, Remark 2.7, since $C\subset \textrm{Fix}T_{\left( \varOmega _{k},\omega _{k}\right) \lambda _{k}}$ and since (by Lemma 4.7) $T_{\left( \varOmega _{k},\omega _{k}\right) \lambda _{k}}$ is $\left( 1+\rho _{\left\{ U_{i}\right\} _{i=1}^{m}}-\lambda _{k}\right) \lambda _{k}^{-1}$-firmly nonexpansive operator (and in particular, a nonexpansive one), we have

4.6 $$\begin{aligned} \left\| x^{k+1}-z\right\| ^{2}&=\left\| T_{\left( \varOmega _{k},\omega _{k}\right) \lambda _{k}}\left( x^{k}\right) -T_{\left( \varOmega _{k},\omega _{k}\right) \lambda _{k}}z\right\| ^{2}\le \left\| x^{k}-z\right\| ^{2}\nonumber \\&-\left( 1+\rho _{\left\{ U_{i}\right\} _{i=1}^{m}}-\lambda _{k}\right) \lambda _{k}^{-1}\left\| \left( x^{k}-T_{\left( \varOmega _{k},\omega _{k}\right) \lambda _{k}}\left( x^{k}\right) \right) \right\| ^{2}\nonumber \\&\le \left\| x^{k}-z\right\| ^{2}-\varepsilon \left( 1+\rho _{\left\{ U_{i}\right\} _{i=1}^{m}}-\varepsilon \right) ^{-1}\left\| \left( x^{k+1}-x^{k}\right) \right\| ^{2}. \end{aligned}$$

As a result, $\left\{ x^{k}\right\} _{k=0}^{\infty }$ is Fejér monotone with respect to *C*. Since the real sequence

$\left\{ \left\| x^{k}-z\right\| ^{2}\right\} _{k=0}^{\infty }$ is monotone decreasing and bounded from below by 0, it converges and (4.6) now implies that $\left\| x^{k+1}-x^{k}\right\| \rightarrow 0$.

*(iii)* Assume that each $T\in I$ is approximately shrinking and that the family $\cap _{T\in I}C_{T}$ is boundedly regular. Let $T\in I$. Clearly, there is a sequence $\left\{ l_{k}\right\} _{k=0}^{\infty }\subset \left\{ 0,1,\dots ,M_{T}-1\right\}$ such that $T=T_{\left( \varOmega _{k+l_{k}},\omega _{k+l_{k}}\right) }$ for each $k\in \mathbb {N}$. Then,

$$ \lambda _{k+l_{k}}\left( T\left( x^{k+l_{k}}\right) -x^{k+l_{k}}\right) =T_{\lambda _{k+l_{k}}}\left( x^{k+l_{k}}\right) -x^{k+l_{k}}=x^{k+l_{k}+1}-x^{k+l_{k}} $$

for each $k\in \mathbb {N}$. This, combined with *(ii)* implies, in its turn (since $\left\{ \lambda _{k+l_{k}}\right\} _{k=0}^{\infty }\subset \left[ \varepsilon ,1+\rho _{\left\{ U_{i}\right\} _{i=1}^{m}}-\varepsilon \right]$), that $\lim _{k\rightarrow \infty }\left\| T\left( x^{k+l_{k}}\right) -x^{k+l_{k}}\right\| =0$. Since *T* is approximately shrinking and the sequence $\left\{ x^{k+l_{k}}\right\} _{k=0}^{\infty }$ is bounded (because it is weakly convergent by *(i)*), it follows that

4.7 $$\begin{aligned} \lim _{k\rightarrow \infty }d\left( x^{k+l_{k}},C_{T}\right) =0. \end{aligned}$$

Now for each $k\in \mathbb {N}$,

4.8 $$\begin{aligned} d\left( x^{k},C_{T}\right) \le \left\| x^{k}-x^{k+l_{k}}\right\| +d\left( x^{k+l_{k}},C_{T}\right) \end{aligned}$$

and

4.9 $$\begin{aligned} \left\| x^{k}-x^{k+l_{k}}\right\| =\left\| \sum _{i=0}^{l_{k}-1}x^{k+i}-x^{k+i+1}\right\| \le \sum _{i=0}^{l_{k}-1}\left\| x^{k+i}-x^{k+i+1}\right\| \end{aligned}$$

(By definition $\sum _{i=0}^{-1}\left\| x^{k+i}-x^{k+i+1}\right\| :=0$). Combining (4.7), (4.8) and (4.9) with *(ii)*, we get, due to the finite number of summands in (4.9), $\lim _{k\rightarrow \infty }d\left( x^{k},C_{T}\right) =0$. Now the bounded regularity of the family $\left\{ C_{T}\right\} _{T\in I}$ implies that $\lim _{k\rightarrow \infty }d\left( x^{k},C\right) =0$. By *(ii)* and Theorem 2.22, the sequence $\left\{ x^{k}\right\} _{k=0}^{\infty }$ converges strongly to a point $x\in C$.

*(iv)* Clearly, $x^{k+1}=T_{\left( \varOmega _{k},\omega _{k}\right) \lambda _{k}}\left( x^{k}\right)$ for each $k\in \mathbb {N}$. Consider bounded perturbations by letting $\left\{ \beta _{k}\right\} _{k=0}^{\infty }$ be a sequence of positive real numbers such that $\sum _{k=0}^{\infty }\beta _{k}<\infty$ and letting $\{v^{k}\}_{k=0}^{\infty }$ be a bounded sequence in $\mathcal {H}$. Assume that $y^{0}\in \mathcal {H}$ and consider the sequence $\left\{ y^{k}\right\} _{k=0}^{\infty }$ generated by the iterative process $y^{k+1}:=T_{\left( \varOmega _{k},\omega _{k}\right) \lambda _{k}}(y^{k}+\beta _{k}v^{k})$. Suppose that $q\in \mathbb {N}$ and $y\in \mathcal {H}$ are arbitrary. For each $k\in \mathbb {N}$, set $\gamma _{k}:=\beta _{k}\left\| v^{k}\right\| \in \left[ 0,\infty \right)$, $\lambda _{k}^{\prime }:=\lambda _{q+k}\in \left[ \varepsilon ,1+\rho _{\left\{ U_{i}\right\} _{i=1}^{m}}-\varepsilon \right]$, $\varOmega _{k}^{\prime }:=\varOmega _{q+k}$ and $\omega _{k}^{\prime }:=\omega _{q+k}$. Since by Remark 4.3, the sequence $\left\{ T_{\left( \varOmega _{k}^{\prime },\omega _{k}^{\prime }\right) }\right\} _{k=0}^{\infty }$ is $\limsup$-admissible and

$$ C=\cap _{T\in \limsup _{k\rightarrow \infty }\left\{ T_{\left( \varOmega _{k},\omega _{k}\right) }\right\} }C_{T}=\cap _{T\in \limsup _{k\rightarrow \infty }\left\{ T_{\left( \varOmega _{k}^{\prime },\omega _{k}^{\prime }\right) }\right\} }C_{T}, $$

it follows from *(i)* that the sequence $\left\{ x^{\prime k}\right\} _{k=0}^{\infty }$ generated by Algorithm 4.5 with respect to the sequences $\left\{ \lambda _{k}^{\prime }\right\} _{k=0}^{\infty }$ and $\left\{ T_{\left( \varOmega _{k}^{\prime },\omega _{k}^{\prime }\right) }\right\} _{k=0}^{\infty }$, where $x^{\prime 0}=y$, converges weakly to a point $x^{\prime }\in C$. Then, for each $k\in \mathbb {N}$, we have

$$ x^{\prime k+1}=T_{\left( \varOmega _{k}^{\prime },\omega _{k}^{\prime }\right) \lambda _{k}^{\prime }}\cdots T_{\left( \varOmega _{1}^{\prime },\omega _{1}^{\prime }\right) \lambda _{1}^{\prime }}T_{\left( \varOmega _{0}^{\prime },\omega _{0}^{\prime }\right) \lambda _{0}^{\prime }}\left( y\right) $$

$$=T_{\left( \varOmega _{q+k},\omega _{q+k}\right) \lambda _{q+k}}\cdots T_{\left( \varOmega _{q+1},\omega _{q+1}\right) \lambda _{q+1}}T_{\left( \varOmega _{q},\omega _{q}\right) \lambda _{q}}\left( y\right) $$

and hence the sequence $\left\{ T_{\left( \varOmega _{q+k},\omega _{q+k}\right) \lambda _{q+k}}\cdots T_{\left( \varOmega _{q+1},\omega _{q+1}\right) \lambda _{q+1}}T_{\left( \varOmega _{q},\omega _{q}\right) \lambda _{q}}\left( y\right) \right\} _{k=0}^{\infty }$ converges weakly to an element of *C* for any arbitrary $y\in \mathcal {H}$. Since for each $k\in \mathbb {N}$, the operator $T_{\left( \varOmega _{k},\omega _{k}\right) \lambda _{k}}$ is nonexpansive, by Lemma 4.7, we obtain

$$\begin{aligned} \left\| y^{k+1}-T_{\left( \varOmega _{k},\omega _{k}\right) \lambda _{k}}\left( y^{k}\right) \right\|&=\left\| T_{\left( \varOmega _{k},\omega _{k}\right) \lambda _{k}}(y^{k}+\beta _{k}v^{k})-T_{\left( \varOmega _{k},\omega _{k}\right) \lambda _{k}}\left( y^{k}\right) \right\| \le \beta _{k}\left\| v^{k}\right\| =\gamma _{k}. \end{aligned}$$

We have,

$$ \cap _{k=0}^{\infty }\textrm{Fix}T_{\left( \varOmega _{k},\omega _{k}\right) \lambda _{k}}=\cap _{k=0}^{\infty }\textrm{Fix}T_{\left( \varOmega _{k},\omega _{k}\right) }=C $$

by Remark 2.7 and since the sequence $\left\{ T_{\left( \varOmega _{k},\omega _{k}\right) }\right\} _{k=0}^{\infty }$ is $\limsup$-admissible. Observe that $\sum _{k=0}^{\infty }\gamma _{k}<\infty$ because the sequence $\{v^{k}\}_{k=0}^{\infty }$ is bounded. We now deduce from Theorem 2.33 that the sequence $\left\{ y^{k}\right\} _{k=0}^{\infty }$ converges weakly to an element of *C* as well, proving that the sequence $\left\{ x^{k}\right\} _{k=0}^{\infty }$ is weakly bounded perturbations resilient with respect to *C*. If the convergence of $\left\{ x^{k}\right\} _{k=0}^{\infty }$ is strong, then, again by Theorem 2.33, $\left\{ x^{k}\right\} _{k=0}^{\infty }$ is strongly bounded perturbations resilient with respect to *C*. This completes the proof of the theorem. $\square$

#### Remark 4.9

- If there exists a $k_{0}\in \mathbb {N}$ such that the sequence $\left\{ T_{\left( \varOmega _{k_{0}+k},\omega _{k_{0}+k}\right) }\right\} _{k=0}^{\infty }$ is $\limsup$-admissible, then by Remark 4.3, the statements of Theorem 4.8 remain true with the only following change in *(ii)*, wherein the sequence $\left\{ x^{k}\right\} _{k=k_{0}}^{\infty }$ (instead of $\left\{ x^{k}\right\} _{k=0}^{\infty }$) is Fejér monotone with respect to *C*, namely,
  $$ \left\| x^{k+1}-z\right\| ^{2}\le \left\| x^{k}-z\right\| ^{2}-\varepsilon \left( 1+\rho _{\left\{ U_{i}\right\} _{i=1}^{m}}-\varepsilon \right) ^{-1}\left\| \left( x^{k+1}-x^{k}\right) \right\| ^{2} $$
  for each natural $k\ge k_{0}$.
- In particular, if $U_{i}$ is a $2\left( \mathcal {M}+1\right) ^{-1}$-relaxed firmly nonexpansive (that is, $\mathcal {M}$-firmly nonexpansive by Theorem 2.12) operator for each $i=1,2,\dots ,m$, then $\rho _{\left\{ U_{i}\right\} _{i=1}^{m}}=1$ and $\lambda _{k}\in \left[ \varepsilon ,2-\varepsilon \right]$ for each $k\in \mathbb {N}$. If $U_{i}$ is a nonexpansive (that is, 2-relaxed firmly nonexpansive by Corollary 2.13) for each $i=1,2,\dots ,m$, then $\rho _{\left\{ U_{i}\right\} _{i=1}^{m}}=0$ and $\lambda _{k}\in \left[ \varepsilon ,1-\varepsilon \right]$ for each $k\in \mathbb {N}$.
- If the space $\mathcal {H}$ is of a finite dimension, then the convergence in *(i)* is strong.

#### Example 4.10

Let $\varepsilon >0$ be a real number. For all $k\in \mathbb {N}$, set $q_{k}:=1$, $\varOmega _{k}:=\left\{ 1,2,\dots ,m\right\} {EMPTY}^{\left\{ 1,2,\dots ,q_{k}\right\} }$ and $\omega _{k}:=\overline{\omega }$ for a fixed $\overline{\omega }:\left\{ 1,2,\dots ,m\right\} {EMPTY}^{\left\{ 1,2,\dots ,q_{k}\right\} }\rightarrow \left( 0,1\right]$. Clearly, for each $i\in \left\{ 1,2,\dots ,m\right\}$, there is a unique string ${t^{i}}\in \left\{ 1,2,\dots ,m\right\} {EMPTY}^{\left\{ 1,2,\dots ,q_{k}\right\} }$ such that $t^{i}\left( 1\right) =i$. Hence we can define the mapping $\omega :\left\{ 1,2,\dots ,m\right\} \rightarrow \left\{ 1,2,\dots ,m\right\} {EMPTY}^{\left\{ 1,2,\dots ,q_{k}\right\} }$ by $\omega _{i}:=\overline{\omega }\left( t^{i}\right)$ for each $i\in \left\{ 1,2,\dots ,m\right\}$. In this case Algorithm 4.5 with the above provisions provides a fully-simultaneous method, that is,

4.10 $$\begin{aligned} T_{\left( \varOmega _{k},\omega _{k}\right) }=\sum _{t\in \varOmega _{k}}\overline{\omega }\left( t\right) U_{t\left( 1\right) }=\sum _{i=1}^{m}\overline{\omega }\left( t^{i}\right) U_{t^{i}\left( 1\right) }=\sum _{i=1}^{m}\omega _{i}U_{i} \end{aligned}$$

for each $k\in \mathbb {N}$, and $C=F=\textrm{Fix}\sum _{i=1}^{m}\omega _{i}U_{i}$. We see from (4.10) that the sequence $\left\{ T_{\left( \varOmega _{k},\omega _{k}\right) }\right\} _{k=0}^{\infty }$ is $\limsup$-admissible. Hence, under the assumption that $\textrm{Fix}\sum _{i=1}^{m}\omega _{i}U_{i}\not =\emptyset$ we obtain, by Theorem 4.8, the weak convergence of this fully-simultaneous method, with parameters $\left\{ \lambda _{k}\right\} _{k=0}^{\infty }\subset \left[ \varepsilon ,1+\rho _{\left\{ U_{i}\right\} _{i=1}^{m}}-\varepsilon \right]$, to a point in *C*. In particular, when $U_{i}=P_{C_{i}}$, where $C_{i}$ is a nonempty, closed and convex subset of $\mathcal {H}$ for each $i=1,2,\dots ,m$ , we obtain the well-known simultaneous projection method, see, for example, [13, Subsection 5.4]. In this case $\rho _{\left\{ U_{i}\right\} _{i=1}^{m}}=1$ (by Example 2.20 and Remark 4.9(b)), $I=\left\{ \sum _{i=1}^{m}\omega _{i}P_{C_{i}}\right\}$ and

4.11 $$\begin{aligned} C=F=\textrm{Fix}\sum _{i=1}^{m}\omega _{i}P_{C_{i}}=\underset{x\in \mathcal {H}}{\textrm{Argmin}}f\left( x\right) , \end{aligned}$$

where $f:\mathcal {H}\rightarrow \mathbb {R}$ is a, so called, proximity function defined by $f:=2^{-1}\sum _{i=1}^{m}\omega _{i}\left\| P_{C_{i}}-Id\right\| ^{2}$. For the proof of the last equality in (4.11), see, for instance, Theorem 4.4.6 in [13]. By Theorem 4.8*(i)*, the simultaneous projection method with parameters $\left\{ \lambda _{k}\right\} _{k=0}^{\infty }\subset \left[ \varepsilon ,2-\varepsilon \right]$ converges weakly to a point in $\underset{x\in \mathcal {H}}{\textrm{Argmin}}f\left( x\right)$. If, in addition, the operator $\sum _{i=1}^{m}\omega _{i}P_{C_{i}}$ is approximately shrinking, then (since the family $\left\{ C_{T}\right\} _{T\in I}=\left\{ C\right\}$ is boundedly regular), this convergence is strong by Theorem 4.8*(ii)*.

## Application of the GDSA Method to the Superiorization Methodology

In this section we introduce the superiorized version of Algorithm 4.5 with respect to a convex and continuous objective function $\phi :\mathcal {H}\rightarrow \mathbb {R}$ and sequences $\left\{ T_{\left( \varOmega _{k},\omega _{k}\right) }\right\} _{n=0}^{\infty }$ and $\left\{ \lambda _{k}\right\} _{k=0}^{\infty }\subset \left[ \varepsilon ,1+\rho _{\left\{ U_{i}\right\} _{i=1}^{m}}-\varepsilon \right]$, where $\varepsilon >0$, defined in Section 4. We investigate its convergence properties in the framework of the Superiorization Methodology. This provides a generalization of Algorithm 4.1, presented in [23] and applies to the results below concerning the behavior of the superiorized version of Algorithm 4.5.

### A few words about the Superiorization Methodology

In this subsection we recall the brief description of the superiorization methodology (SM), quoted from the preface to the 2017 special issue [20] on “Superiorization: Theory and Applications”. “The superiorization methodology is used for improving the efficacy of iterative algorithms whose convergence is resilient to certain kinds of perturbations. Such perturbations are designed to “force” the perturbed algorithm to produce more useful results for the intended application than the ones that are produced by the original iterative algorithm. The perturbed algorithm is called the “superiorized version” of the original unperturbed algorithm. If the original algorithm is computationally efficient and useful in terms of the application at hand and if the perturbations are simple and not expensive to calculate, then the advantage of this method is that, for essentially the computational cost of the original algorithm, we are able to get something more desirable by steering its iterates according to the designed perturbations. This is a very general principle that has been used successfully in some important practical applications, especially for inverse problems such as image reconstruction from projections, intensity-modulated radiation therapy and nondestructive testing, and awaits to be implemented and tested in additional fields.”

Further information and references on the SM can be found in papers listed in the bibliographic collection on the dedicated Webpage [16]. For recent works that include introductory material on the SM see, for example, [18, 25, 26] and [30].

### The Superiorized Version of the GDSA Algorithm

In Subsection 4.1 we defined $I:=\limsup _{k\rightarrow \infty }\left\{ T_{\left( \varOmega _{k},\omega _{k}\right) }\right\}$ and the family $\left\{ C_{T}\right\} _{T\in I}$ with the intersection $C:=\cap _{T\in I}C_{T}$. Let $\phi :\mathcal {H}\rightarrow \mathbb {R}$ be a convex and continuous real valued objective function. Define the constrained minimum set by

$$ C_{\min }:=\textrm{Argmin}\{\phi \left( x\right) \mid x\in C\}. $$

#### Algorithm 5.1

(The superiorized version of the GDSA algorithm) Given $y^{0}\in \mathcal {H}$, a sequence $\left\{ N_{k}\right\} _{k=0}^{\infty }$ of positive integers, a sequence $\left\{ \lambda _{k}\right\} _{k=0}^{\infty }$ of positive numbers and a family of positive real sequences $\left\{ \left\{ \beta _{k,n}\right\} _{n=1}^{N_{k}}\right\} _{k=0}^{\infty }$ such that $\sum _{k=0}^{\infty }\sum _{n=1}^{N_{k}}\beta _{k,n}<\infty$, the algorithm is defined by the recurrences

$$ y^{k+1}:=y^{k}+\sum _{n=1}^{N_{k}}\beta _{k,n}v^{k,n}+\lambda _{k}\left( T_{\left( \varOmega _{k},\omega _{k}\right) }\left( y^{k}+\sum _{n=1}^{N_{k}}\beta _{k,n}v^{k,n}\right) -y^{k}-\sum _{n=1}^{N_{k}}\beta _{k,n}v^{k,n}\right) $$

$$ \textrm{wherein} $$

5.1 $$\begin{aligned} v^{k,n+1}:={\left\{ \begin{array}{ll} -\left\| s^{k,n}\right\| ^{-1}s^{k,n}, &  \textrm{if}\,\,0\not \in \partial \phi \left( y^{k}+\sum _{i=1}^{n}\beta _{k,i}v^{k,i}\right) ,\\ 0, &  \textrm{if}\,\,0\in \partial \phi \left( y^{k}+\sum _{i=1}^{n}\beta _{k,i}v^{k,i}\right) , \end{array}\right. } \end{aligned}$$

for each $k\in \mathbb {N}$ and each $n=0,1,\dots ,N_{k}-1$, where $s^{k,n}$ is a selection of the subgradient $\partial \phi \left( y^{k}+\sum _{i=1}^{n}\beta _{k,i}v^{k,i}\right)$ (which exists by Theorem 2.34) for each $k\in \mathbb {N}$ and each $n=0,1,\dots ,N_{k}-1$ (recalling that, by definition, $\sum _{i=1}^{0}\beta _{k,i}v^{k,i}:=0$).

It is true that, under the assumptions of Theorem 4.8, where, in particular, $\left\{ \lambda _{k}\right\} _{k=0}^{\infty }\subset \left[ \varepsilon ,1+\rho _{\left\{ U_{i}\right\} _{i=1}^{m}}-\varepsilon \right]$ for $\varepsilon >0$, the sequence $\left\{ y^{k}\right\} _{k=0}^{\infty }$, generated by Algorithm 5.1 with respect to the sequence $\left\{ \lambda _{k}\right\} _{k=0}^{\infty }$, satisfies Statements *(i)* and *(iii)* of Theorem 4.8. Indeed, define a sequence $\left\{ \beta _{k}\right\} _{k=0}^{\infty }$ of positive real numbers by $0<\beta _{k}:=\sum _{n=1}^{N_{k}}\beta _{k,n}$ for each $k\in \mathbb {N}$. Then, $\sum _{k=0}^{\infty }\beta _{k}<\infty$ and we have

5.2 $$\begin{aligned} y^{k+1}&=T_{\left( \varOmega _{k},\omega _{k}\right) \lambda _{k}}\left( y^{k}+\sum _{n=1}^{N_{k}}\beta _{k,n}v^{k,n}\right) =T_{\left( \varOmega _{k},\omega _{k}\right) \lambda _{k}}\left( y^{k}+\beta _{k}\sum _{n=1}^{N_{k}}\beta _{k,n}\beta _{k}^{-1}v^{k,n}\right) . \end{aligned}$$

It follows, by the triangle inequality and (5.1) that

$$ \left\| \sum _{n=1}^{N_{k}}\beta _{k,n}\beta _{k}^{-1}v^{k,n}\right\| \le \sum _{n=1}^{N_{k}}\beta _{k,n}\beta _{k}^{-1}=1, $$

that is, the sequence $\left\{ \sum _{n=1}^{N_{k}}\beta _{k,n}\beta _{k}^{-1}v^{k,n}\right\} _{k=0}^{\infty }$ is bounded in $\mathcal {H}$. By using Theorem 4.8*(iv)* and (5.2), we obtain the following corollary.

#### Corollary 5.2

Let $y^{0}\in \mathcal {H}$. Assume that the sequence $\left\{ T_{\left( \varOmega _{k},\omega _{k}\right) }\right\} _{k=0}^{\infty }$ is $\limsup$-admissible and $C\not =\emptyset$. Let $\left\{ \lambda _{k}\right\} _{k=0}^{\infty }$ be a sequence of real numbers such that $\lambda _{k}\in \left[ \varepsilon ,1+\rho _{\left\{ U_{i}\right\} _{i=1}^{m}}-\varepsilon \right]$ for each $k\in \mathbb {N}$, where $\varepsilon >0$. Then the sequence $\left\{ y^{k}\right\} _{k=0}^{\infty }$ generated by Algorithm 5.1 converges weakly to a point $y\in C$. If, in addition, each $T\in I$ is approximately shrinking and the family $\left\{ C_{T}\right\} _{T\in I}$ is boundedly regular, then $\left\{ y^{k}\right\} _{k=0}^{\infty }$ converges strongly to a point $y\in C$.

It turns out that if the sequence $\left\{ y^{k}\right\} _{k=0}^{\infty }$, generated by Algorithm 5.1, converges strongly to a point $y\in \mathcal {H}$, then the sequence $\left\{ y^{k}\right\} _{k=k_{0}}^{\infty }$ is strictly Fejér monotone with respect to $C_{\textrm{min}}$ for some $k_{0}\in \mathbb {N}$. In order to show this, we need the following auxiliary lemma.

#### Lemma 5.3

For an arbitrary nonempty subset *C* of $\mathcal {H}$ and $y,z\in C$ such that $z\in \textrm{Argmin}_{x\in C}\phi \left( x\right)$ and $y\not \in \textrm{Argmin}_{x\in C}\phi \left( x\right)$, there exist real numbers $r_{1}>0$ and $r_{2}>0$ so that for each $\overline{y}\in B\left( y,r_{1}\right)$ and $v\in \partial \phi \left( \overline{y}\right)$, the following assertions are satisfied:

(i) $0\not \in \partial \phi \left( \overline{y}\right)$ and for each $\overline{z}\in B\left( z,r_{2}\right)$
  5.3 $$\begin{aligned} \left\langle \left\| v\right\| ^{-1}v,\overline{z}-\overline{y}\right\rangle <0. \end{aligned}$$
(ii) We have
  5.4 $$\begin{aligned} \left\langle \left\| v\right\| ^{-1}v,z-\overline{y}\right\rangle <-2^{-1}r_{2}. \end{aligned}$$
(iii) Let *p* be a nonnegative integer. Assume that $\left\{ \alpha _{n}\right\} _{n=1}^{p}$ is a sequence of positive real numbers such that $\sum _{n=1}^{p}a_{n}<2^{-1}r_{1}$ and $\left\{ v^{n}\right\} _{n=1}^{p}\subset \mathcal {H}\backslash \left\{ 0\right\}$ is a sequence such that $v^{n}\in \partial \phi \left( \overline{y}-\sum _{i=1}^{n-1}\alpha _{i}\left\| v^{i}\right\| ^{-1}v^{i}\right)$ for each $n=1,2,\dots ,p$. If, in addition, $\overline{y}\in B\left( y,2^{-1}r_{1}\right)$, then
  5.5 $$\begin{aligned} \left\| \overline{y}-\sum _{n=1}^{p}\alpha _{n}\left\| v^{n}\right\| ^{-1}v^{n}-z\right\| ^{2}\le \left\| \overline{y}-z\right\| ^{2}-\sum _{n=1}^{p}\left( r_{2}-\alpha _{n}\right) \alpha _{n} \end{aligned}$$
  (by definition $$\sum _{n=1}^{0}\alpha _{n}\left\| v^{n}\right\| ^{-1}v^{n}:=\sum _{n=1}^{0}\left( r_{2}-\alpha _{n}\right) \alpha _{n}:=0)$$.

#### Proof

Since $z\in \textrm{Argmin}_{x\in C}\phi \left( x\right)$ and $y\not \in \textrm{Argmin}_{x\in C}\phi \left( x\right)$, we observe that $\phi \left( y\right) -\phi \left( z\right) >0$. By the continuity of $\phi$, there exist $r_{1}>0$ and $r_{2}>0$ such that

5.6 $$\begin{aligned} \phi \left( \overline{y}\right) -\phi \left( \overline{z}\right) >0, \end{aligned}$$

for each $\overline{y}\in B\left( y,r_{1}\right)$ and $\overline{z}\in B\left( z,r_{2}\right)$. Let $\overline{y}\in B\left( y,r_{1}\right)$ and $v\in \partial \phi \left( \overline{y}\right)$.

*(i)* In view of (5.6) and (2.1), we have for each $\overline{z}\in B\left( z,r_{2}\right)$,

$$ \left\langle v,\overline{z}-\overline{y}\right\rangle <0. $$

It follows that $v\not =0$ and (5.3) holds. Since *v* is an arbitrary element of $\partial \phi \left( \overline{y}\right)$, it follows that $0\not \in \partial \phi \left( \overline{y}\right)$.

*(ii)* Set $\overline{z}:=z+2^{-1}r_{2}\left\| v\right\| ^{-1}v$. Then $\overline{z}\in B\left( z,r_{2}\right)$ and by (5.3),

$$ \left\langle \left\| v\right\| ^{-1}v,z+2^{-1}r_{2}\left\| v\right\| ^{-1}v-\overline{y}\right\rangle =\left\langle \left\| v\right\| ^{-1}v,\overline{z}-\overline{y}\right\rangle <0 $$

and (5.4) follows.

*(iii)* Assume that $\overline{y}\in B\left( y,2^{-1}r_{1}\right)$. Since $\sum _{n=1}^{p-1}a_{n}<2^{-1}r_{1}$, we obtain

5.7 $$\begin{aligned} \left( \overline{y}-\sum _{n=1}^{p-1}\alpha _{n}\left\| v^{n}\right\| ^{-1}v^{n}\right) \in B\left( y,r_{1}\right) . \end{aligned}$$

It is true that,

5.8 $$\begin{aligned} v^{p}\in \partial \phi \left( \overline{y}-\sum _{n=1}^{p-1}\alpha _{n}\left\| v^{n}\right\| ^{-1}v^{n}\right) . \end{aligned}$$

The proof of (5.5) is by induction on *p*. Clearly, (5.5) is true for $p=0$. Assume that $p>0$. Then by the induction hypothesis, (5.7), (5.8) and *(ii)* above,

$$\begin{aligned} \left\| \overline{y}-\sum _{n=1}^{p}\alpha _{n}\left\| v^{n}\right\| ^{-1}v^{n}-z\right\| ^{2}&=\left\| \overline{y}-\sum _{n=1}^{p-1}\alpha _{n}\left\| v^{n}\right\| ^{-1}v^{n}-\alpha _{p}\left\| v^{p}\right\| ^{-1}v^{p}-z\right\| ^{2}\\&=\left\| \overline{y}-\sum _{n=1}^{p-1}\alpha _{n}\left\| v^{n}\right\| ^{-1}v^{n}-z\right\| ^{2}\\&+2\alpha _{p}\left\langle \left\| v^{p}\right\| ^{-1}v^{p},z-\left( \overline{y}-\sum _{n=1}^{p-1}\alpha _{n}\left\| v^{n}\right\| ^{-1}v^{n}\right) \right\rangle +\alpha _{p}^{2}\\&\le \left\| \overline{y}-z\right\| ^{2}-\sum _{n=1}^{p-1}\left( r_{2}-\alpha _{n}\right) \alpha _{n}-\alpha _{p}r_{2}+\alpha _{p}^{2}\\&=\left\| \overline{y}-z\right\| ^{2}-\sum _{n=1}^{p}\left( r_{2}-\alpha _{n}\right) \alpha _{n}. \end{aligned}$$

Lemma 5.3 is now proved. $\square$

The following “theorem of alternatives” provides a more general analogue of Theorem 4.1 in [23] in the setting of our superiorized version of the GDSA algorithm.

#### Theorem 5.4

Let $y^{0}\in \mathcal {H}$ and assume that the sequence $\left\{ y^{k}\right\} _{k=0}^{\infty }$, generated by Algorithm 5.1 with respect to the sequence $\left\{ \lambda _{k}\right\} _{k=0}^{\infty }\subset \left[ \varepsilon ,1+\rho _{\left\{ U_{i}\right\} _{i=1}^{m}}-\varepsilon \right]$, where $\varepsilon >0$, converges strongly to a point $y\in C$. Then exactly one of the following two alternatives holds:

(i) $$y\in C_{\textrm{min}}$$.
  or
(ii) $y\not \in C_{\textrm{min}}$ and there exists $k_{0}\in \mathbb {N}$ such that $\left\{ y^{k}\right\} _{k=k_{0}}^{\infty }$ is strictly Fejér monotone with respect to $C_{\textrm{min}}$. Namely, there exists a sequence $\left\{ u_{k}\right\} _{k=k_{0}}^{\infty }$ of positive real numbers such that $\left\| y^{k+1}-z\right\| ^{2}\le \left\| y^{k}-z\right\| ^{2}-u_{k}$ for every $z\in C_{\textrm{min}}$ and for all natural $k\ge k_{0}$.

#### Proof

Assume that $\left\{ y^{k}\right\} _{k=0}^{\infty }$ converges strongly to a point $y\not \in C_{\textrm{min}}$. Then, $y\in C$ by Corollary 5.2 and $y\not \in \textrm{Argmin}_{x\in C}\phi \left( x\right)$. Assume that $z\in C_{\textrm{min}}$. By Lemma 5.3, there exist real numbers $r_{1}>0$ and $r_{2}>0$ such that each $\overline{y}\in B\left( y,r_{1}\right)$ and $v\in \partial \phi \left( \overline{y}\right)$ satisfy its assertions. By using the strong convergence of $\left\{ y^{k}\right\} _{k=0}^{\infty }$ to *y* and the convergence of the series $\sum _{k=0}^{\infty }\sum _{n=1}^{N_{k}}\beta _{k,n}$, choose $k_{0}\in \mathbb {N}$ such that

5.9 $$\begin{aligned} y^{k}\in B\left( y,2^{-1}r_{1}\right) \end{aligned}$$

and

5.10 $$\begin{aligned} \sum _{n=1}^{N_{k}}\beta _{k,n}<\min \left\{ 2^{-1}r_{1},r_{2}\right\} \end{aligned}$$

for each integer $k\ge k_{0}$. This yields, for each $k\ge k_{0}$,

$$ y^{k}+\sum _{i=1}^{n-1}\beta _{k,i}v^{k,i}\in B\left( y,r_{1}\right) $$

for each $n=1,2,\dots ,N_{k}$, and, consequently, by Lemma 5.3*(i)*,

5.11 $$\begin{aligned} 0\not \in \partial \phi \left( y^{k}+\sum _{i=1}^{n-1}\beta _{k,i}v^{k,i}\right) \end{aligned}$$

for each $n=1,2,\dots ,N_{k}$. Let $k\ge k_{0}$ be an integer. By (5.1) and (5.11),

5.12 $$\begin{aligned} v^{k,n}=-\left\| s^{k,n-1}\right\| ^{-1}s^{k,n-1}, \end{aligned}$$

where

5.13 $$\begin{aligned} s^{k,n-1}\in \partial \phi \left( y^{k}+\sum _{i=1}^{n-1}\beta _{k,i}v^{k,i}\right) , \end{aligned}$$

for each $n=1,2,\dots ,N_{k}$. Set $p:=N_{k}$ and $\overline{y}:=y^{k}$. For each $n=1,2,\dots ,p$, define $\alpha _{n}:=\beta _{k,n}>0$ and $v^{n}:=s^{k,n-1}$. Then, by (5.9), (5.10), (5.11), (5.12) and (5.13), $\overline{y}\in B\left( y,2^{-1}r_{1}\right)$, $\sum _{n=1}^{p}\alpha _{n}<2^{-1}r_{1}$, $\left\{ v^{n}\right\} _{n=1}^{p}\subset \mathcal {H}\backslash \left\{ 0\right\}$ and $v^{n}\in \partial \phi \left( \overline{y}-\sum _{i=1}^{n-1}\alpha _{i}\left\| v^{i}\right\| ^{-1}v^{i}\right)$ for each $n=1,2,\dots ,p$. Since the operator $T_{\left( \varOmega _{k},\omega _{k}\right) \lambda _{k}}$, the $\lambda _{k}$-relaxation of $T_{\left( \varOmega _{k},\omega _{k}\right) }$, is nonexpansive, by Lemma 4.7, we obtain from Lemma 5.3*(iii),* that

$$\begin{aligned} \left\| y^{k+1}-z\right\| ^{2}&=\left\| T_{\left( \varOmega _{k},\omega _{k}\right) \lambda _{k}}\left( y^{k}+\sum _{n=1}^{N_{k}}\beta _{k,n}v^{k,n}\right) -z\right\| ^{2}\le \left\| y^{k}+\sum _{n=1}^{N_{k}}\beta _{k,n}v^{k,n}-z\right\| ^{2}\\&=\left\| \overline{y}-\sum _{n=1}^{p}\alpha _{n}\left\| v_{n}\right\| ^{-1}v_{n}-z\right\| ^{2}\le \left\| \overline{y}-z\right\| ^{2}-\sum _{n=1}^{p}\left( r_{2}-\alpha _{n}\right) \alpha _{n}\\&=\left\| y^{k}-z\right\| ^{2}-\sum _{n=1}^{N_{k}}\left( r_{2}-\beta _{k,n}\right) \beta _{k,n}. \end{aligned}$$

Now set $u_{k}:=\sum _{n=1}^{N_{k}}\left( r_{2}-\beta _{k,n}\right) \beta _{k,n}$ for each natural $k\ge k_{0}$. Then since $\sum _{n=1}^{N_{k}}\beta _{k,n}<r_{2}$, by (5.10), the result follows and the proof of the theorem is complete. $\square$

#### Remark 5.5

Note that we do not assume any admissibility condition on the sequence of operators $\left\{ T_{\left( \varOmega _{k},\omega _{k}\right) }\right\} _{k=0}^{\infty }$ in Theorem 5.4.

Combining Theorem 5.4 with Corollary 5.2, we obtain the following corollary.

#### Corollary 5.6

Let $y^{0}\in \mathcal {H}$ and assume that the sequence $\left\{ T_{\left( \varOmega _{k},\omega _{k}\right) }\right\} _{k=0}^{\infty }$ is $\limsup$-admissible. Suppose also that each $T\in I$ is approximately shrinking and the family $\left\{ C_{T}\right\} _{T\in I}$ is boundedly regular. Then the sequence $\left\{ y^{k}\right\} _{k=0}^{\infty }$ generated by Algorithm 5.1 with respect to the sequence $\left\{ \lambda _{k}\right\} _{k=0}^{\infty }\subset \left[ \varepsilon ,1+\rho _{\left\{ U_{i}\right\} _{i=1}^{m}}-\varepsilon \right]$, where $\varepsilon >0$, converges strongly to a point $y\in C$ and exactly one of the following two alternatives holds:

(i) $$y\in C_{\textrm{min}}$$
  or
(ii) $y\not \in C_{\textrm{min}}$ and there exists $k_{0}\in \mathbb {N}$ such that $\left\{ y^{k}\right\} _{k=k_{0}}^{\infty }$ is strictly Fejér monotone with respect to $C_{\textrm{min}}$. Namely, there exists a sequence $\left\{ u_{k}\right\} _{k=k_{0}}^{\infty }$ of positive real numbers such that for every $z\in C_{\textrm{min}}$, $\left\| y^{k+1}-z\right\| ^{2}\le \left\| y^{k}-z\right\| ^{2}-u_{k}$ for all natural $k\ge k_{0}$.

## Conclusion

In this paper we proposed and investigated a General Dynamic String-Averaging (GDSA) iterative scheme in the inconsistent case. The main tool is the property called “strong coherence” which serves as a sufficient condition for convergence of iterative schemes governed by infinite sequences of operators. The GDSA algorithm is bounded perturbation resilient and, as such, we applied to it the superiorization methodology and derived for the superiorized version of the GDSA algorithm a “theorem of alternatives” proving strict Fejér monotonicity with respect to the minimum set of the underlying constrained minimization problem data.

## Data Availability

No data was used for the research described in the article.
